# Brain Atrophy Does Not Predict Clinical Progression in Progressive Supranuclear Palsy

**DOI:** 10.1002/mds.70026

**Published:** 2025-08-30

**Authors:** Andrea Quattrone, Nicolai Franzmeier, Hans‐Jürgen Huppertz, Nicholas Seneca, Gabor C. Petzold, Annika Spottke, Johannes Levin, Johannes Prudlo, Emrah Düzel, the PASSPORT Study Group, the AL‐108‐231 Investigators, the Arise Investigators, the Tauros MRI Investigators, the DESCRIBE‐PSP group, Ikuko Aiba, Angelo Antonini, Diana Apetauerova, Jean‐Philippe Azulay, Ernest Balaguer Martinez, Jee Bang, Paolo Barone, Matthew Barrett, Danny Bega, Daniela Berg, Koldo Berganzo Corrales, Yvette Bordelon, Adam L Boxer, Moritz Brandt, Norbert Brueggemann, Giovanni Castelnovo, Roberto Ceravolo, Rosalind Chuang, Sun Ju Chung, Alistair Church, Jean‐Christophe Corvol, Paola Cudia, Marian Dale, Luc Defebvre, Sophie Drapier, Erika D Driver‐Dunckley, Georg Ebersbach, Karla M Eggert, Aaron Ellenbogen, Alexandre Eusebio, Andrew H Evans, Natalia Fedorova, Elizabeth Finger, Alexandra Foubert‐Samier, Boyd Ghosh, Lawrence Golbe, Francisco Grandas Perez, Murray Grossman, Deborah Hall, Kyoko Hamada, Kazuko Hasegawa, Guenter Hoeglinger, Lawrence Honig, David Houghton, Xuemei Huang, Stuart Isaacson, Seong‐Beom Koh, Jaime Kulisevsky Bojarski, Anthony E. Lang, Peter Nigel Leigh, Irene Litvan, Juan Jose Lopez Lozano, Jose Luis Lopez‐Sendon Moreno, Albert Christian Ludolph, Ma Rosario Luquin Piudo, Irene Martinez Torres, Nikolaus McFarland, Wassilios Meissner, Tiago Mestre, Pablo Mir Rivera, Eric Molho, Britt Mollenhauer, Huw R Morris, Miho Murata, Tomokazu Obi, Fabienne Ory Magne, Padraig O'Suilleabhain, Rajesh Pahwa, Alexander Pantelyat, Nicola Pavese, Dmitry Pokhabov, Johannes Prudlo, Federico Rodriguez‐Porcel, James Rowe, Joseph Savitt, Alfons Schnitzler, Joerg B Schulz, Klaus Seppi, Binit Shah, Holly Shill, David Shprecher, Maria Stamelou, Malcolm Steiger, Yuji Takahashi, Hiroshi Takigawa, Carmela Tartaglia, Lars Toenges, Daniel Truong, Winona Tse, Paul Tuite, Dieter Volc, Anne‐Marie A Wills, Dirk Woitalla, Tao Xie, Tatsuhiko Yuasa, Sarah Elizabeth Zauber, Theresa Zesiewicz, David Williams, Anne Louise Lafontaine, Connie Marras, Mandar Jog, Michael Panisset, Anthony Lang, Lesley Parker, Alistair J. Stewart, Jean‐Christophe Corvol, Jean‐Philippe Azulay, Philippe Couratier, Brit Mollenhauer, Stefan Lorenzl, Albert Ludolph, Reiner Benecke, Günter Höglinger, Axel Lipp, Heinz Reichmann, Dirk Woitalla, Dennis Chan, Adam Zermansky, David Burn, Andrew Lees, Adam Boxer, Bruce L. Miller, Iryna V. Lobach, Erik Roberson, Lawrence Honig, Edward Zamrini, Rajesh Pahwa, Yvette Bor‐delon, Erika Driver‐Dunkley, Stephanie Lessig, Mark Lew, Kyle Womack, Brad Boeve, Joseph Ferrara, Argyle Hillis, Daniel Kaufer, Rajeev Kumar, Tao Xie, Steven Gunzler, Theresa Zesiewicz, Praveen Dayalu, Lawrence Golbe, Murray Grossman, Joseph Jancovic, Scott McGinnis, Anthony Santiago, Paul Tuite, Stuart Isaacson, Julie Leegwater‐Kim, Irene Litvan, Murray Grossman, David S. Knopman, Bruce L. Miller, Lon S. Schneider, Rachelle S. Doody, Lawrence I. Golbe, Erik D. Roberson, Mary Koestler, Clifford R. Jack, Viviana Van Deerlin, Christopher Randolph, Iryna V. Lobach, Illana Gozes, Steve Whitaker, Joe Hirman, Michael Gold, Bruce H. Morimoto, Anwar Ahmed, Ikuko Aiba, Alberto Albanese, Kelly Bertram, Yvette Bordelon, James Bower, Jared Brosch, Daniel Claassen, Carlo Colosimo, Jean‐Christophe Corvol, Paola Cudia, Antonio Daniele, Luc Defebvre, Erika Driver‐Dunckley, Antoine Duquette, Roberto Eleopra, Alexandre Eusebio, Victor Fung, David Geldmacher, Lawrence Golbe, Francisco Grandas, Deborah Hall, Taku Hatano, Günter U Höglinger, Lawrence Honig, Jennifer Hui, Diana Kerwin, Akio Kikuchi, Thomas Kimber, Takashi Kimura, Rajeev Kumar, Irene Litvan, Peter Ljubenkov, Stefan Lorenzl, Albert Ludolph, Zoltan Mari, Nikolaus McFarland, Wassilios Meissner, Pablo Mir Rivera, Hidek Mochizuki, John Morgan, Renato Munhoz, Noriko Nishikawa, John O'Sullivan, Tomoko Oeda, Hideki Oizumi, Osamu Onodera, Fabienne Ory‐Magne, Elizabeth Peckham, Ronald Postuma, Aldo Quattrone, Joseph Quinn, Stefano Ruggieri, Justyna Sarna, Paul E. Schulz, John Slevin, Michele Tagliati, Daryl Wile, Zbigniew Wszolek, Tao Xie, Theresa Zesiewicz, Juan C. Gómez, Beatriz Tijero, Rafael Villoria, Justo García de Yebenes, Jose L. Sendón, Eduardo Tolosa, Maria T. Buongiorno, Nuria Bargalló, Juan A. Burguera, Iñiguez Martinez, Javier Ruiz‐Martínez, Jorge Villanua, Francisco Vivancos, Isabel Ybot, Miquel Aguilar, Josep L. Dolz, Mercè Boada, Asuncion Lafuente, Miguel A. Tejero, Juan J. López‐Lozano, Marina Mata, Andreas Kupsch, Axel Lipp, Matthias Höllerhage, Wolfgang H. Oertel, Gesine Respondek, Maria Stamelou, Susanne Knake, Daniela Berg, Walter Maetzler, Karin K. Srulijes, Adriane Gröger, Albert Ludolph, Jan Kassubek, Malcom Steiger, Kevin Tyler, David J. Burn, Cristopher Morris, Andrew Lees, Helen Ling, Lisa Strycharczuk, Slawek Altenstein, Mathias Baehr, Miriam Barkhoff, Claudia Bartels, Bartl Michael, Rudi Beschorner, Eberhard Karls, Aline Beyle, Henning Boecker, Moritz Brandt, Kathrin Brockmann, Frederic Brosseron, Lena Burow, Cihan Catak, Arda Can Cetindag, Nicoleta Carmen Cosma, Marcel Daamen, Eman Dashti, Sylvia de Jonge, Peter Dechent, Martin Dichgans, Elisabeth Dinter, Laura Dobisch, Angelika Dörr, Markus Donix, Louisa Droste zu Senden, Alexander Drzezga, Emrah Düzel, Martin Dyrba, Marie Ehrlich, Matthias Endres, Tanja Engels, Ersin Ersözlü, Michael Ewers, Jennifer Faber, Björn Falkenburger, Frederike Fenski, Klaus Fließbach, Agnes Flöel, Silka Dawn Freiesleben, Franca Laura Fries, Ingo Frommann, Thomas Gasser, Wenzel Glanz, Doreen Goerss, Doreen Grieger‐Klose, Marcus Grobe‐Einsler, Martin Grond, Selim Üstün Gürsel, Annett Halle, Niels Hansen, Deike Hartmann, Lina Hassoun, Robert Haussmann, Julian Hellmann‐Regen, Guido Hennes, Wiebke Hermann, Andreas Hermann, Jochen Herms, Gabi Herrmann, Stefan Hetzer, Petra Hinderer, Günther U. Höglinger, Ildiko Horvath, Nicole Hujer, Enise Irem Incesoy, Alexander Jäck, Daniel Janowitz, Frank Jessen, Lorraine Jost, Pascal Kalbhen, Jan Kassubek, Sabrina Katzdobler, Ingo Kilimann, Okka Kimmich, Luca Kleinadam, Thomas Klockgether, Xenia Kobeleva, Patricia Krause, Elke Kuder‐Buletta, Andrea Kühn, Carolin Kurz, Catharina Lange, Chris Lappe, Christoph Laske, Johannes Levin, Sandra Lichte Schneider, Paul Lingor, Matthias Löhle, Albert C. Ludolph, Falk Lüsebrink, Cornelia McCormick, David Mengel, Coraline Metzger, Matthias Munk, Manuela Neumann, Demet Oender, Robert Perneczky, Oliver Peters, Gabor C. Petzold, Henrike Pfaff, Alexandra Polcher, Lukas Preis, Josef Priller, Johannes Prudlo, Dominik Pürner, Veronika Purrer, Boris Rauchmann, Alfredo Ramirez, Heike Raum, Sandra Röske, Christin Ruß, Petr Sabik, Yilmaz Sagik, Klaus Scheffler, Monika Schmidt, Anja Schneider, Luisa‐Sophie Schneider, Ludger Schoels, Björn Schott, Heike Schulz, Franziska Schulze, Sebastian Sodenkamp, Annika Spottke, Eike Spruth, Melina Stark, Matthis Synofzik, Patricia Sulzer, Stefan Teipel, Ina R Vogt, Michael Wagner, Endy Weidinger, Carlo Wilke, Jens Wiltfang, Steffen Wolfsgruber, Ullrich Wüllner, Renat Yakupov, Heike Zech, Inga Zerr, Gabriel Ziegler, Adelgunde Zollver, Günter U. Höglinger

**Affiliations:** ^1^ Department of Neurology University Hospital, LMU Munich Munich Germany; ^2^ Institute of Neurology, Department of Medical and Surgical Sciences Magna Graecia University Catanzaro Italy; ^3^ Neuroscience Research Centre Magna Graecia University Catanzaro Italy; ^4^ Institute for Stroke and Dementia Research (ISD), University Hospital, LMU Munich Germany; ^5^ Munich Cluster for Systems Neurology (SyNergy) Munich Germany; ^6^ Department of Psychiatry and Neurochemistry University of Gothenburg, The Sahlgrenska Academy, Institute of Neuroscience and Physiology Mölndal and Gothenburg Sweden; ^7^ Swiss Epilepsy Clinic, Klinik Lengg Zurich Switzerland; ^8^ Precision Medicine—Neuroscience, AbbVie Inc North Chicago Illinois USA; ^9^ German Center for Neurodegenerative Diseases (DZNE) Bonn Germany; ^10^ Division of Vascular Neurology, Department of Neurology University Hospital Bonn Bonn Germany; ^11^ Department of Neurology University of Bonn Bonn Germany; ^12^ German Center for Neurodegenerative Diseases (DZNE) Munich Munich Germany; ^13^ German Center for Neurodegenerative Diseases (DZNE) Rostock‐Greifswald Germany; ^14^ Department of Neurology University Medical Centre Rostock Germany; ^15^ German Center for Neurodegenerative Diseases (DZNE) Magdeburg Germany; ^16^ Institute of Cognitive Neurology and Dementia Research Otto‐von‐Guericke University Magdeburg Germany; ^17^ Institute of Cognitive Neuroscience University College London London United Kingdom

**Keywords:** progressive supranuclear palsy, atlas‐based volumetry, outcome, progression, clinical trials

## Abstract

**Background:**

Clinical progression rate is the typical primary endpoint measure in progressive supranuclear palsy (PSP) clinical trials.

**Objectives:**

This longitudinal multicohort study investigated whether baseline clinical severity and regional brain atrophy could predict clinical progression in PSP–Richardson's syndrome (PSP‐RS).

**Methods:**

PSP‐RS patients (n = 309) from the placebo arms of clinical trials (NCT03068468, NCT01110720, NCT02985879, NCT01049399) and DescribePSP cohort were included. We investigated associations of baseline clinical and volumetric magnetic resonance imaging (MRI) data with 1‐year longitudinal PSP rating scale (PSPRS) change. Machine learning (ML) models were tested to predict individual clinical trajectories.

**Results:**

PSP‐RS patients showed a mean PSPRS score increase of 10.3 points/yr. The frontal lobe volume showed the strongest association with subsequent clinical progression (β: −0.34, *P* < 0.001). However, ML models did not accurately predict individual progression rates (*R* ^2^ <0.15).

**Conclusions:**

Baseline clinical severity and brain atrophy could not predict individual clinical progression, suggesting no need for MRI‐based stratification of patients in future PSP trials. © 2025 The Author(s). *Movement Disorders* published by Wiley Periodicals LLC on behalf of International Parkinson and Movement Disorder Society.

Progressive supranuclear palsy–Richardson's syndrome (PSP‐RS) is a neurodegenerative disease characterized by brain 4R‐tau accumulation and atrophy.^1^, ^2^ Several clinical trials have been carried out in PSP, measuring clinical disease progression as a primary end point to evaluate treatment efficacy.^3^, ^4^, ^5^, ^6^ A major challenge in advancing research on disease‐modifying therapies, however, is the heterogeneity of clinical progression rates across patients. PSP includes several clinical phenotypes,^1^ which typically show divergent clinical trajectories, with more rapid progression and shorter survival in PSP‐RS than in other variants.^7^, ^8^, ^9^, ^10^, ^11^ Moreover, although PSP‐RS patients typically show a mean change of 9–11 points/yr in the PSP rating scale (PSPRS),^3^, ^4^, ^5^, ^6^, ^7^, ^12^, ^13^, ^14^, ^15^, ^16^ variability exists, and individuals may differ considerably from this group‐derived average rate. This heterogeneity may affect clinical trial results, as hypothetical random differences in natural history decline among the two trial arms might exacerbate or mask drug efficacy. Baseline information on the future clinical trajectory of each patient would enable a precise baseline stratification and easy detection of deviations from the individual natural disease course. By leveraging longitudinal large‐scale data from the placebo arms of several clinical trials^3^, ^4^, ^5^, ^6^, ^7^ and a German observational study,^17^ the current multicohort study aimed to investigate if baseline clinical severity and regional brain atrophy were associated at the group level with longitudinal clinical progression, and whether machine learning (ML) models may predict individual 1‐year clinical progression in PSP‐RS patients.

## Materials and Methods

PSP‐RS patients from the placebo arms of randomized controlled trials (NCT03068468,^3^ NCT01110720,^4^ NCT02985879,^5^ NCT01049399)^6^ and the DescribePSP network cohort^17^ were included. A multicohort group of 258 age‐matched healthy controls (HC) described in a previous study^18^ was used for calculating percentage change and *z* scores of imaging data in PSP patients. The study population was based on these inclusion criteria: (1) probable PSP‐RS diagnosis according to the MDS criteria,^1^ (2) available demographic data (sex, age), (3) available PSPRS^20^ scores at baseline and 1‐year follow‐up, (4) available baseline T_1_‐weighted brain magnetic resonance imaging (MRI). MR images were processed through automated atlas‐based volumetry, as previously described.^18^, ^19^ Volumes were summed for bilateral regions and normalized to intracranial volume. Detailed procedures on patient cohorts and MRI processing are provided in Supporting Information.

## Statistics and Machine Learning

Comparisons between groups were performed using Fisher's, Wilcoxon, analysis of variance (ANOVA), or Kruskal‐Wallis test, with Bonferroni correction. Clinical progression was measured as annualized PSPRS absolute or percentage change over time, as described in Supporting Information. Associations between baseline variables and clinical progression were investigated using linear regression models (covariates: age, sex), evaluating regression coefficients (*R* ^2^) and false discovery rate (FDR)‐adjusted *P*‐values. Subsequently, we employed ML technology to predict individual clinical progression using baseline clinical and/or radiological data. We employed support vector machine (SVM) with RBF kernel,^19^ and a tree‐based classifier (Random Forest [RF]).^21^ We used two alternative approaches: a regression task to predict the individual annualized PSPRS total score change and a classification task to distinguish “fast progressors” from “slow progressors” (stratified by the median annualized PSPRS absolute or percentage change values). Performance metrics included the *R* ^2^ (regression tasks) and area under the receiver operating characteristic curve (AUC‐ROC) (classification tasks), calculated on unseen data through fivefold stratified nested cross‐validation procedure. Further details are provided in Supporting Information.

## Results

The cohort included 309 PSP‐RS patients (Fig. S1). All subcohorts from different trials were comparable for age, sex, and clinical severity at baseline and follow‐up visits (Table 1). PSP‐RS patients showed ventricular enlargement and widespread brain atrophy at baseline, most prominent in the midbrain, red nucleus, subthalamic nucleus, substantia nigra, superior cerebellar peduncles (SCPs), and globus pallidus (Table S1). At baseline, volumes of several brain structures showed weak but significant associations with baseline clinical severity (PSPRS total score). The strongest associations were found for SCPs (regression‐derived β: −0.36), brainstem and third ventricle (β: −0.34), midbrain, pons and medulla (β: −0.32), and inferior cerebellar peduncle (β: −0.31), all with FDR‐corrected *P* < 0.001 (Table S2).

**TABLE 1:** Demographic and clinical data of the study participants

Data	All PSP patients (n = 309)	Gosuranemab cohort (n = 133)	Davunetide cohort (n = 98)	Tilavonemab cohort (n = 52)	DescribePSP cohort (n = 22)	*P*‐value across PSP cohorts
Sex (M/F)	161/148	72/61	46/52	29/23	12/10	0.658^a^
Age (yr)	68.8 ± 6.5	69.6 ± 6.4	67.3 ± 7.0	68.6 ± 5.5	71.0 ± 6.9	0.032^b^ ^,^ *
PSPRS total score	37.6 ± 10.3	37.3 ± 9.5	38.7 ± 10.9	36.2 ± 9.7	38.1 ± 13.3	0.721^b^
PSPRS history score	7.9 ± 3.2	7.7 ± 2.8	8.3 ± 3.4	7.4 ± 3.6	8.1 ± 3.3	0.356^b^
PSPRS mentation score	3.7 ± 2.5	3.6 ± 2.2	3.9 ± 2.8	3.5 ± 2.4	4.0 ± 2.9	0.777^b^
PSPRS bulbar score	2.8 ± 1.5	2.8 ± 1.4	2.7 ± 1.5	2.5 ± 1.3	3.1 ± 1.9	0.422^b^
PSPRS ocular score	8.9 ± 3.1	8.6 ± 3.1	9.4 ± 2.9	9.0 ± 3.0	7.7 ± 3.2	0.091^b^
PSPRS limb score	4.6 ± 2.0	4.8 ± 2.0	4.6 ± 1.9	4.3 ± 1.7	4.6 ± 2.6	0.540^b^
PSPRS gait and midline score	9.8 ± 3.7	9.8 ± 3.5	9.8 ± 3.9	9.5 ± 3.7	10.5 ± 4.2	0.927^b^
SEADL	56.8 ± 20.7	57.4 ± 20.0	56.5 ± 22.5	57.1 ± 20.3	53.2 ± 19.6	0.813^b^
*Longitudinal data*						
Inter‐visit interval (mo)	12.0 ± 0.5	12.0 ± 0.2	12.0 ± 0.0	11.8 ± 0.5	13.0 ± 1.5	**<0.001** ^b^ ^,^ ^#^
PSPRS total score at follow‐up	47.9 ± 13.2	47.6 ± 13.6	48.5 ± 13.1	46.8 ± 11.9	49.8 ± 15.2	0.785^b^
Annualized PSPRS total score absolute change	10.3 ± 8.9	10.3 ± 8.6	9.86 ± 9.7	10.8 ± 8.6	10.8 ± 9.0	0.813^b^
Annualized PSPRS total score percentage change	29.8 ± 28.0	28.6 ± 26.4	28.9 ± 29.0	33.9 ± 30.8	32.2 ± 29.6	0.813^b^

*Note*: The Tideglusib cohort included 4 patients only and is not shown in the table. Significant ANOVA or Kruskal‐Wallis test *P*‐values surviving FDR correction are highlighted in bold. Data are shown as mean ± standard deviation.

Abbreviations: PSP, progressive supranuclear palsy; PSPRS, PSP rating scale; SEADL, Schwab and England Activities of Daily Living scale.

* No differences in post hoc analyses.

^#^ All pairwise comparisons showed significant *P*‐values in post hoc analyses (after the Bonferroni correction), except for the Davunetide cohort versus Gosuranemab cohort comparison.

^a^ Fisher's exact test.

^b^ ANOVA or Kruskal‐Wallis rank‐sum test followed by pairwise *t* test or Wilcoxon rank‐sum test.

### Associations between Baseline Data and Longitudinal Clinical Progression

**FIG. 1:**
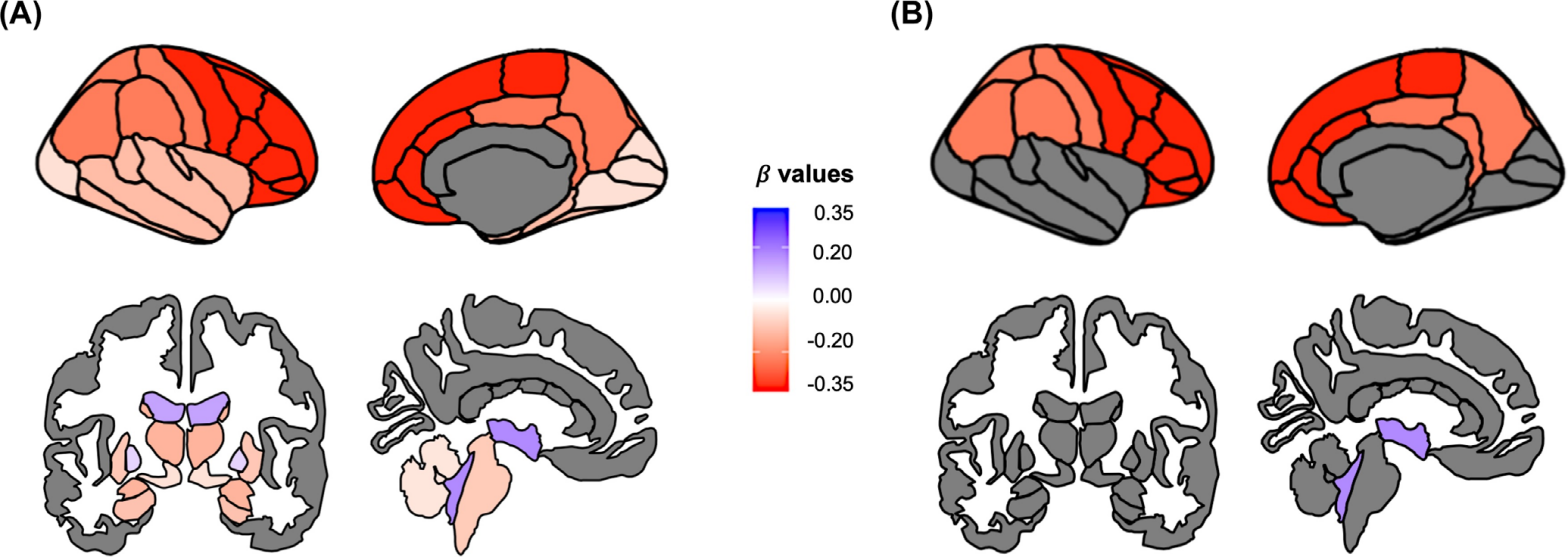
The figure shows baseline regional volumes of cortical and subcortical structures associated with future 1‐year clinical progression rate as measured by the annualized absolute change in the PSP rating scale total score. All associations are shown on the left (**A**), whereas only significant associations with false discovery rate (FDR)‐adjusted *P‐*value <0.05 are shown on the right (**B**). The color scale represents the association strength as measured by the linear regression‐derived β value, with shades of red for negative associations (lower volume associated with faster progression) and shades of blue for positive associations (larger volume associated with faster progression). All models also included age and sex as follows: (annualized absolute change in the PSP rating scale ~ regional volume + age + sex). Please note that some structures (ie, cerebellar peduncles) are not shown in the figure; full data are shown in the tables. [Color figure can be viewed at wileyonlinelibrary.com]

PSP‐RS patients showed significant progression in clinical scores over 1‐year time (baseline vs. follow‐up visit, *P* < 0.001), including SEADL, PSPRS total score, and all its categories. The PSPRS total score showed a mean annualized increase of 29.8% (10.3 points), and the progression rate was nearly identical across all subcohorts from different trials (Figs. S2 and S3). At the group level, several baseline regional volumes were associated with longitudinal clinical progression measured as annualized PSPRS score absolute change, with higher baseline atrophy corresponding to faster subsequent clinical decline (Fig. 1; Table S3). The strongest association was found for the frontal lobe (β: −0.34, *P* < 0.001) (Figs. 1 and S4). Age, sex, and baseline clinical severity were not associated with absolute change in PSPRS score. Conversely, when the percentage, rather than absolute PSPRS change, was considered, all baseline PSPRS categories and total score were associated with progression (milder baseline severity corresponding to faster progression, Table S3; Fig. S4). Baseline volumes of whole brain, cerebrospinal fluid (CSF), frontal lobe and parietal lobe confirmed their association with clinical progression in this analysis (Table S3).

### Individual Longitudinal Trajectory Prediction Modeling

All the linear models described above, including a single baseline variable, explained <15% of variance in the longitudinal PSPRS score change across patients (*R* ^2^ < 0.15). Suboptimal results were obtained also by linear models, including multiple clinical and/or volumetric data (adjusted *R* ^2^ < 0.25, Table S4). Subsequently, we employed ML algorithms (SVM and RF) aiming to predict individual clinical progression in PSP‐RS patients using baseline data, using both regression and classification approaches. In the regression tasks, both algorithms showed *R* ^2^ < 0.15 in the validation folds in predicting annualized absolute (or percentage) change in PSPRS score. Representative plots showing real and predicted PSPRS change values are shown in Figure S5. Similarly, in the classification tasks, both algorithms provided AUC‐ROC values <0.70 in distinguishing fast from slow progressors in the validation folds (Table S5). Overall, ML models yielded largely suboptimal results in predicting individual clinical progression rates in PSP‐RS patients.

## Discussion

This study demonstrated that PSP‐RS patients have variable clinical progression rates over 1 year. Patients with more atrophic frontal lobe volume at baseline visit had faster clinical progression over the following year, but neither baseline severity nor imaging regional volumetric data allowed to accurately predict individual trajectories.

In this multicohort study, we observed a mean increase of 10.3 point (29.8%) in the PSP rating scale, providing robust natural‐history data in a large international population of PSP‐RS patients eligible for trials. This progression rate was nearly identical across PSP‐RS subcohorts from the placebo arms of previous trials, demonstrating that these groups were homogeneous for severity and progression, and can be merged for future cross‐sectional or longitudinal research. A surprising insight was that around 10% of patients clinically improved over time despite PSP‐RS being a progressive disease. Possible explanations include occasional misdiagnosis, day‐to‐day fluctuations in clinical symptoms, different physicians evaluating the patient at baseline and follow‐up assessments, and placebo effects. Beyond these few atypical cases, PSP‐RS patients showed great heterogeneity in individual progression rates, ranging from 0 to 40–50‐point increase in the PSPRS over 1 year. Thus, we investigated the relevance of baseline clinical severity and regional brain atrophy as predictors of future clinical progression. Regarding clinical severity, we found negative associations with the annualized percentage change in the PSPRS score, but not with its absolute change. This discrepancy was possibly explained because many patients showed 10‐to‐20‐point PSPRS score longitudinal increase, but this corresponded to variable percentage score changes depending on the baseline severity. This finding is relevant for trial design, supporting the use of PSPRS absolute rather than percentage change as clinical end point, which may require smaller sample size to detect treatment effect,^22^ and is less influenced by baseline severity.

Regarding brain atrophy, patients with more severe brain atrophy had worse clinical symptoms and progressed faster in the subsequent year. Brainstem structures showed the highest atrophy degree and the strongest associations with clinical severity at baseline, whereas cortical structures (frontal and parietal lobes) were more associated with subsequent clinical progression. In particular, the frontal lobe volume (showing mild atrophy at baseline) was the most associated imaging variable with subsequent decline, with faster progression in patients displaying more severe baseline atrophy. A similar phenomenon was observed in a recent study,^23^ where the midbrain and cerebellum correlated with disease severity at baseline, whereas other regions (nucleus accumbens and putamen) showed stronger correlations with survival. A possible explanation may be that midbrain is early affected in PSP‐RS,^24^ and is already atrophic when patients undergo their first MRI scan, correlating with clinical severity but not providing information on future progression. Conversely, the cortical or striatal involvement occurs later,^24^ and these structures are atrophic only in a patient subset at the stage they are enrolled in trials; thus, patients who already display frontal lobe atrophy likely have more widespread neurodegenerative processes compatible with faster symptom progression in the following months. These results expand the current knowledge on features associated with clinical progression in PSP‐RS, currently including clinical scales,^14^, ^25^ MRI,^14^, ^26^, ^27^, ^28^ positron emission tomography (PET) data,^25^, ^29^ and fluid biomarkers.^30^, ^31^ In addition, we first investigated whether these group associations may translate into individual predictions using ML technology. We employed RF and SVM models to estimate the disease progression in individual patients using baseline clinicoradiological data, but the models did not explain more than 15% of variability in progression rates across subjects and failed in distinguishing “fast progressors” from “slow progressors” (AUC‐ROC <0.70). Overall, these data demonstrate limited usefulness of structural MRI and baseline severity for patient stratification in terms of future progression. In this study, we used robust and well‐known ML models (SVM, RF), which are commonly employed in research as they are relatively easy to implement at limited computational cost. This approach can facilitate comparisons with other data sources (diffusion imaging, PET, fluid biomarkers, etc.), which may be more informative to this aim. For the sake of completeness, an eXtreeme Gradient Boosting (XGB) and an ensemble stacking model combining SVM, RF, and XGB were also tested (data not shown), obtaining very similar results. However, artificial intelligence technology is rapidly evolving, and future studies are warranted to investigate whether advanced deep learning or data fusion strategies might overcome the limitations of current approaches.

This study has several strengths. First, the large multicohort of PSP‐RS patients ensure generalizability of the results. Second, our cohort mainly included patients from PSP clinical trials, thus generating evidence useful for future trial design. Third, all MRI measures were obtained using automated operator‐independent volumetry. Fourth, we employed ML algorithms based on mathematically different prediction methods, and the results were consistent, providing a solid basis for justifying the conclusions. Among limitations, we focused on patients fulfilling inclusion criteria of previous trials, typically consistent with PSP‐RS phenotype^7^, ^32^; thus PSP variants were not included. Second, baseline PET and fluid biomarker data (ie, neurofilament light chains or exosome protein levels^33^) were not available; integrating different biomarkers may lead to better prediction of progression rates, as demonstrated in the Alzheimer's field,^34^ and future studies addressing this point are warranted. Third, although we measured clinical progression using the PSPRS change, we did not include outcome measures as milestones or survival.

Overall, this study demonstrated that regional brain atrophy was associated with 1‐year clinical progression at the group level but had limited potential in predicting individual trajectories. These findings may be relevant for trial design, suggesting that MRI‐based stratification of patients at baseline may not significantly impact the trial results in PSP‐RS.

## Author Roles

(1) Research project: A. Conception, B. Organization, C. Execution; (2) Statistical analysis: A. Design, B. Execution, C. Review and critique; (3) Manuscript preparation: A. Writing of the first draft, B. Review and critique.

A.Q.: 1A, 1C, 2A, 2B, 3A

N.F.: 1A, 1B, 2C, 3B

H.‐J.H.: 1C, 3B

N.S.: 1B, 3B

G.C.P.: 1B, 3B

A.S.: 1B, 3B

J.L.: 1B, 3B

J.P.: 1B, 3B

E.D.: 1B, 3B

G.U.H.: 1A, 1B, 2C, 3B

## Disclosures

**Funding Sources and Conflicts of Interest:** Andrea Quattrone received funding from the Italian Ministry of Health, not related to the current research. Hans‐Jürgen Huppertz has used atlas‐based volumetric MRI analysis in industry‐sponsored research projects. Johannes Levin reports speaker fees from Bayer Vital, Biogen, EISAI, TEVA, Zambon, Esteve, Merck and Roche; consulting fees from Axon Neuroscience, EISAI, and Biogen; author fees from Thieme medical publishers and W. Kohlhammer GmbH medical publishers and is inventor of a patent “Oral Phenylbutyrate for Treatment of Human 4‐Repeat Tauopathies” (PCT/EP2024/053388) filed by LMU Munich. In addition, he reports compensation for serving as chief medical officer for MODAG GmbH, is beneficiary of the phantom share program of MODAG GmbH, and is inventor of a patent “Pharmaceutical Composition and Methods of Use” (EP 22159408.8) filed by MODAG GmbH, all activities outside the submitted work. Günter Höglinger participated in industry‐sponsored research projects from AbbVie, Biogen, Biohaven, Novartis, Roche, Sanofi, UCB; has ongoing research collaborations with Roche, UCB, AbbVie; serves as a consultant for AbbVie, Alzprotect, Amylyx, Aprineua, Asceneuron, Bayer, Bial, Biogen, Biohaven, Epidarex, Ferrer, Kyowa Kirin, Lundbeck, Novartis, Retrotope, Roche, Sanofi, Servier, Takeda, Teva, UCB; received honoraria for scientific presentations from AbbVie, Bayer, Bial, Biogen, Bristol Myers Squibb, Kyowa Kirin, Pfizer, Roche, Teva, UCB, Zambon. Günter Höglinger was funded by the Deutsche Forschungsgemeinschaft (DFG, German Research Foundation) under Germany's Excellence Strategy within the framework of the Munich Cluster for Systems Neurology (EXC 2145 SyNergy—ID 390857198), European Joint Programme on Rare Diseases (Improve‐PSP), Niedersächsisches Ministerium für Wissenschaft und Kunst (MWK)/VolkswagenStiftung (Niedersächsisches Vorab), Petermax‐Müller Foundation (Etiology and Therapy of Synucleinopathies and Tauopathies), the German Parkinson Society (DPG): ProAPS. The other authors declared no conflicts of interest.

## Supporting information


**Supplementary Figure S1.** A flowchart showing the study inclusion/exclusion procedures. Progressive supranuclear palsy–Richardson's syndrome (PSP‐RS) patients with available longitudinal clinical data, including the PSP rating scale score and available baseline brain magnetic resonance imaging (MRI), were selected from the placebo arms of clinical trials and from the DescribePSP cohort observational study. The final patient cohort included 309 PSP‐RS patients.

**Supplementary Figure S2.** (**A**) The left part of the figure includes histograms showing the annualized PSPRS total score absolute change in PSP‐RS patients in the whole study cohort (red) and in the largest subcohorts from different trials (green, blue, and violet). In each plot, the black dotted line represents the median value, which is also shown in **B**. The right part of the figure includes density plots of both percentage and absolute PSPRS total score change value distributions, allowing easy visual comparison of clinical progression rates across cohorts. PSP‐RS, progressive supranuclear palsy–Richardson's syndrome; PSPRS, PSP rating scale.

**Supplementary Figure S3.** Histograms showing the annualized PSPRS total score percentage change in PSP‐RS patients in the whole study cohort (red) and in the largest subcohorts from different trials (green, blue, and violet). In each plot, the black dotted line represents the median value, which is also shown in the figure. PSP‐RS, progressive supranuclear palsy–Richardson's syndrome; PSPRS, PSP rating scale.

**Supplementary Figure S4.** Scatterplots showing the strongest associations of baseline clinical and imaging features with longitudinal clinical progression as measured by PSP rating scale (PSPRS). On the left, the associations between baseline frontal lobe volume (in mL) and the annualized longitudinal PSPRS total absolute (**A**) or percentage score change (**C**); on the right, the association between baseline PSPRS total score and the annualized longitudinal PSPRS total score absolute (**B**) or percentage change (**D**). The blue color was used for significant associations, and the red color for nonsignificant associations.

**Supplementary Figure S5.** Scatterplots showing on *x*‐axis the real annualized PSP‐RS total score absolute change, and on the *y*‐axis the values predicted by support vector regression (**A**) or random forest regression (**B**) models in the validation folds using baseline clinical and volumetric data. Black dotted lines show the ideal fit.

**Data S1.** Supporting information.

**Supplementary Table S1.** Demographic and imaging data of patients with progressive supranuclear palsy and control subjects.

**Supplementary Table S2.** Associations between regional brain volumes and clinical severity at baseline in patients with progressive supranuclear palsy.

**Supplementary Table S3.** Associations of baseline clinical and imaging data with longitudinal clinical progression in patients with progressive supranuclear palsy.

**Supplementary Table S4.** Explained interpatient variance of clinical progression by multivariate linear regression modeling using clinical and/or imaging data.

**Supplementary Table S5.** Classification performance of machine learning models in differentiating fast from slow progressors in the validation folds.

## Data Availability

The data that support the findings of this study are available from the owners of data, that were collected in clinical trials. Restrictions apply to the availability of these data, which were used under license for this study. Data are available from the author(s) with the permission of the owners of data, that were collected in clinical trials.

## Appendix A

The members of the study groups are listed below:

### A.1 PASSPORT Study Group

Ikuko Aiba (National Hospital Organization Higashinagoya National Hospital, Nagoya, Aichi, Japan), Angelo Antonini (San Camillo Hospital IRCCS, Venice Lido, Italy), Diana Apetauerova (Lahey Hospital and Medical Center, Burlington, MA, USA), Jean‐Philippe Azulay (Assistance Publique Hapitaux De Marseille, Marseille, France), Ernest Balaguer Martinez (Hospital General de Catalunya, Barcelona, Spain), Jee Bang (The Johns Hopkins University, Baltimore, MD, USA), Paolo Barone (University of Salerno, Salerno, Italy), Matthew Barrett (University of Virginia Health System, Charlottesville, VA, USA), Danny Bega (Northwestern University, Chicago, IL, USA), Daniela Berg (UKSH—Campus Kiel, Kiel, Germany), Koldo Berganzo Corrales (Hospital De Cruces, Barakaldo, Spain), Yvette Bordelon (University of California, Los Angeles, CA, USA), Adam L Boxer (Memory and Aging Center, Department of Neurology, University of California, San Francisco, CA, USA), Moritz Brandt (Universitatsklinikum Carl Gustav Carus Dresden, Dresden, Germany), Norbert Brueggemann (University Hospital Schleswig‐Holstein, Luebeck, Germany), Giovanni Castelnovo (Centre Hospitalier Universitaire de Nimes—Hopital Universitaire Caremeau, Nimes, France), Roberto Ceravolo (University Hospital of Pisa, Pisa, Italy), Rosalind Chuang (Swedish Health Services, Seattle, WA, USA), Sun Ju Chung (Asan Medical Center, Seoul, Republic of Korea), Alistair Church (Aneurin Bevan University Health Board—Clinical Research and Innovation Centre—St Woolos Hospital, Newport, UK), Jean‐Christophe Corvol (Sorbonne Université, Assistance Publique Hôpitaux de Paris, INSERM, CNRS, Institut du Cerveau—Paris Brain Institute—ICM, Hôpital Pitié‐Salpêtrière, Paris, France), Paola Cudia (Biogen, Cambridge, MA, USA), Marian Dale (Medical University of South Carolina, Charleston, SC, USA), Luc Defebvre (Centre Hospitalier Regional Universitaire) de Lille—Hopital Roger Salengro, Lille, France), Sophie Drapier (CHU de Rennes—Hopital de Pontchaillou, Rennes, France), Erika D Driver‐Dunckley (Mayo Clinic Arizona—Scottsdale, Scottsdale, AZ, USA), Georg Ebersbach (Movement Disorders Clinic, Beelitz‐Heilstatten, Germany), Karla M. Eggert (Philipps Universitat Marburg, Marburg, Germany), Aaron Ellenbogen (QUEST Research Institute, Farmington Hills, MI, USA), Alexandre Eusebio (Assistance Publique Hapitaux De Marseille, Marseille, France), Andrew H. Evans (The Royal Melbourne Hospital (RMH)‐Flemington Neurology—North Melbourne, North Melbourne, Australia), Natalia Fedorova (Russian Medical Academy of Postgraduate Education, Moscow, Russia), Elizabeth Finger (Parkwood Institute, London, Ontario, Canada), Alexandra Foubert‐Samier (CHU De Bordeaux Parkinson Expert Centre, IMNC Hopital Pellegrin, Bordeaux, France), Boyd Ghosh (University Hospital Southampton NHS Foundation Trust, Southampton UK), Lawrence Golbe (Rutgers Robert Wood Johnson Medical School, New Brunswick, NJ, USA), Francisco Grandas Perez (Hospital General Universitario Gregorio Maranon, Madrid, Spain), Murray Grossman (Hospital of the University of Pennsylvania, Philadelphia, PA, USA), Deborah Hall (Rush University Medical Centre Chicago, IL USA), Kyoko Hamada (Shinsapporo Neurosurgical Hospital, Sapporo, Japan), Kazuko Hasegawa (National Hospital Organization Sagamihara National Hospital, Sagamihara, Japan), Guenter Hoeglinger (University Hospital, Ludwig‐Maximilians‐Universität [LMU] Munich, Munich, Germany), Lawrence Honig (Columbia University College of Physicians and Surgeons—Gertrude H. Sergievsky Center, New York, NY, USA), David Houghton (Ochsner Medical Center, New Orleans, LA, USA), Xuemei Huang (Penn State University‐Milton S. Hershey Medical Center, Hershey, PA, USA), Stuart Isaacson (Parkinson's Disease and Movement Disorder Center of Boca Raton, Boca Raton, FL, USA), Seong‐Beom Koh (Korea University Guro Hospital, Seoul, Republic of Korea), Jaime Kulisevsky Bojarski (Hospital de la Santa Creu i Sant Pau, Barcelona, Spain), Anthony E. Lang (Edmond J. Safra Program in Parkinson's Disease and the Rossy PSP Centre, Toronto Western Hospital and the University of Toronto, Toronto, Ontario, Canada), Peter Nigel Leigh (Brighton and Sussex Medical School Trafford Centre for Biomedical Research, Brighton, UK), Irene Litvan (University of California, Parkinson and Other Movement Disorders Center, San Diego, CA, USA), Juan Jose Lopez Lozano (Clinica Ruber Internacional, Madrid, Spain), Jose Luis Lopez‐Sendon Moreno (Hospital Universitario Ramon y Cajal, Madrid, Spain), Albert Christian Ludolph (Universitats und Rehabilitationskliniken Ulm, Ulm, Germany), Ma Rosario Luquin Piudo (Clinica Universidad De Navarra, Pamplona, Spain), Irene Martinez Torres (Hospital la FE, Valencia, Spain), Nikolaus McFarland (University of Florida Center for Movement Disorders and Neurorestoration, Gainesville, FL, USA), Wassilios Meissner (CHU De Bordeaux Parkinson Expert Centre, IMNC Hopital Pellegrin, Bordeaux, France), Tiago Mestre (The Ottawa Hospital—Civic Campus, University of Ottawa, Ottawa, Canada), Pablo Mir Rivera (Hospital Universitario Virgen del Rocio, Sevilla, Spain), Eric Molho (Albany Medical College, Albany, NY, USA), Britt Mollenhauer (Paracelsus‐Elena‐Klinik Kassel, Kassel, Germany), Huw R. Morris (National Hospital for Neurology and Neurosurgery, London, UK), Miho Murata (Musashi Hospital, Kodaira‐Shi, Japan), Tomokazu Obi (National Hospital Organization—Shizuoka Institute of Epilepsy and Neurological Disorders, Shizuoka, Japan), Fabienne Ory Magne (Hopital Purpan—Batiment Pierre Paul Riquet, Toulouse, France), Padraig O'Suilleabhain (University of Texas Southwestern Medical Center—Clinical Center for Movement Disorders, Dallas, TX, USA), Rajesh Pahwa (The University of Kansas Medical Center—Parkinson's Disease and Movement Disorder Center, Kansas City, KS, USA), Alexander Pantelyat (The Johns Hopkins University, Baltimore, MD, USA), Nicola Pavese (The Newcastle upon Tyne Hospitals NHS Foundation Trust—Campus for Ageing and Vitality, Newcastle upon Tyne, UK), Dmitry Pokhabov (Federal State Budgetary Institution, Federal Siberian Scientific Clinical Center of Federal Medical‐Biological Agency, Krasnoyarsk, Russia), Johannes Prudlo (Universitaet Rostock—Universitaetsmedizin Rostock, Rostock, Germany), Federico Rodriguez‐Porcel (Medical University of South Carolina, Charleston, SC, USA), James Rowe (Cambridge University, Cambridge, UK), Joseph Savitt (University of Maryland School of Medicine, Baltimore, MD, USA), Alfons Schnitzler (Center for Movement Disorders and Neuromodulation‐University Hospital Dusseldorf, Dusseldorf, Germany), Joerg B. Schulz (Uniklinik RWTH Aachen Medizinische Klinik III, Aachen, Germany), Klaus Seppi (Medizinische Universitaet Innsbruck, Innsbruck, Austria), Binit Shah (University of Virginia Health System, Charlottesville, VA, USA), Holly Shill (St. Joseph's Hospital and Medical Center/Barrow Neurology Clinics, Phoenix, AZ, USA), David Shprecher (Banner Sun Health Research Institute, Sun City, AZ, USA), Maria Stamelou (Hygeia Hospital, Marousi, Greece), Malcolm Steiger (The Walton Center—NHS Foundation Trust, Liverpool, UK), Yuji Takahashi (Musashi Hospital, Kodaira‐Shi, Japan), Hiroshi Takigawa (Tottori University Hospital, Yonago, Japan), Carmela Tartaglia (Toronto Western Hospital, University Health Network Movement Disorders Centre, Toronto, Canada), Lars Toenges (St. Josef—Hospital Bochum, Kardiologische Studienambulanz, Bochum, Germany), Daniel Truong (The Parkinson's and Movement Disorder Institute, Fountain Valley, CA, USA), Winona Tse (Mount Sinai Movement Disorders Center, New York, NY, USA), Paul Tuite (University of Minnesota Medical Center—Fairview—Neurology Clinic, Minneapolis, MN, USA), Dieter Volc (Prosenex Ambulatoriumsbetriebs GmbH—Studienzentrum, Vienna, Austria), Anne‐Marie A. Wills (Massachusetts General Hospital Cancer Center, Boston, MA, USA), Dirk Woitalla (St. Josef‐ Krankenhaus, Essen‐Kupferdreh, Essen, Germany), Tao Xie (The University of Chicago Medicine—Center for Parkinson's Disease and Movement Disorders, Chicago, IL, USA), Tatsuhiko Yuasa (Kamagaya General Hospital, Kamagaya, Japan), Sarah Elizabeth Zauber (Indiana University Health Physicians—Neurology—Indianapolis, Indianapolis, IN, USA), Theresa Zesiewicz (University of South Florida—Morsani College of Medicine, Tampa, FL, USA).

### A.2 The AL‐108‐231 Study Group

Australia: David Williams.

Canada: Anne Louise Lafontaine, Connie Marras, Mandar Jog, Michael Panisset, Anthony Lang, Lesley Parker, Alistair J. Stewart.

France: Jean‐Christophe Corvol, Jean‐Philippe Azulay, Philippe Couratier.

Germany: Brit Mollenhauer, Stefan Lorenzl, Albert Ludolph, Reiner Benecke, Günter Höglinger, Axel Lipp, Heinz Reichmann, Dirk Woitalla.

United Kingdom: Dennis Chan, Adam Zermansky, David Burn, Andrew Lees.

United States: Adam Boxer, Bruce L. Miller, Iryna V. Lobach, (Memory and Aging Center, Department of Neurology, University of California, San Francisco, CA, USA), Erik Roberson, Lawrence Honig, Edward Zamrini, Rajesh Pahwa, Yvette Bor‐delon, Erika Driver‐Dunkley, Stephanie Lessig, Mark Lew, Kyle Womack, Brad Boeve, Joseph Ferrara, Argyle Hillis, Daniel Kaufer, Rajeev Kumar, Tao Xie, Steven Gunzler, Theresa Zesiewicz, Praveen Dayalu, Lawrence Golbe, Murray Grossman, Joseph Jancovic, Scott McGinnis, Anthony Santiago, Paul Tuite, Stuart Isaacson, Julie Leegwater‐Kim, Irene Litvan, Murray Grossman, David S. Knopman, Bruce L. Miller, Lon S. Schneider, Rachelle S. Doody, Lawrence I. Golbe, Erik D. Roberson, Mary Koestler, Clifford R. Jack, Jr., Viviana Van Deerlin, Christopher Randolph, Iryna V. Lobach, Illana Gozes, Steve Whitaker, Joe Hirman, Michael Gold, Bruce H. Morimoto.

### A.3 Arise Investigators

Anwar Ahmed (Cleveland Clinic Main Campus, Cleveland, OH), Ikuko Aiba (National Hospital Organization (NHO) Higashinagoya National Hospital, Nagoya‐shi, Japan), Alberto Albanese (IBD Center—Istituto Di Ricovero e Cura a Carattere Scientifico (IRCCS) Istituto Clinico Humanitas, Rozzano, Italy), Kelly Bertram (Alfred Hospital, Melbourne, Australia), Yvette Bordelon (University of California, Los Angeles [UCLA], Los Angeles, CA), James Bower (Mayo Clinic—Rochester, Rochester, MN), Jared Brosch (Indiana University, Indianapolis, IN), Daniel Claassen (Vanderbilt University Medical Center, Nashville, TN), Carlo Colosimo (Azienda Ospedaliera [AO] Santa Maria, Terni, Italy), Jean‐Christophe Corvol (Hopital Pitie Salpetriere, Paris, France), Paola Cudia (Istituto Di Ricovero e Cura a Carattere Scientifico [IRCCS] Ospedale San Camillo, Venezia Lido, Italy), Antonio Daniele (IRCCS) Fondazione Policlinico Universitario A. Gemelli Università Cattolica del Sacro Cuore, Rome, Italy), Luc Defebvre (Hopital B Roger Salengro, Lille, France), Erika Driver‐Dunckley (Mayo Clinic—Scottsdale, Scottsdale, AZ), Antoine Duquette (Centre de recherche du Centre hospitalier de l'Université de Montréal [CRCHUM], Montreal, Canada), Roberto Eleopra (Fondazione IRCCS Istituto Neurologico Carlo Besta, Milan, Italy), Alexandre Eusebio (Hopital de la Timone, Marseille, France), Victor Fung (Westmead Hospital, Westmead, Australia), David Geldmacher (University of Alabama, Birmingham, AL), Lawrence Golbe (Rutgers Robert Wood Johnson University Hospital, New Brunswick, NJ), Francisco Grandas (Hospital General Universitario Gregorio Marañón, Madrid, Spain), Deborah Hall (Rush University Medical Center, Chicago, IL), Taku Hatano (Juntendo University Hospital, Bunkyo‐ku, Japan), Günter U Höglinger (Neurologie, Klinikum rechts der Isar, München, Munich, Germany), Lawrence Honig (Columbia University Medical Center, New York, NY), Jennifer Hui (University of Southern California [USC], Los Angeles, CA), Diana Kerwin (Kerwin Research Center, Dallas, TX), Akio Kikuchi (Tohoku University Hospital, Sendai‐shi, Japan), Thomas Kimber (Royal Adelaide Hospital, Adelaide, Australia), Takashi Kimura (National Hospital Organization Asahikawa Medical Center, Asahikawa, Japan), Rajeev Kumar (Rocky Mountain Movement Disorders Center, Englewood, CO), Irene Litvan (University of California San Diego [UCSD], La Jolla, CA), Peter Ljubenkov (University of California, San Francisco [UCSF], San Francisco, CA), Stefan Lorenzl (Krankenhaus Agatharied GmbH, Hausham, Germany), Albert Ludolph (Universitaetsklinikum Ulm, Ulm, Germany), Zoltan Mari (Cleveland Clinic—Lou Ruvo Center for Brain Health, Las Vegas, NV), Nikolaus McFarland (University of Florida, Gainesville, FL), Wassilios Meissner (Centre Hospitalier Universitaire [CHU] de Bordeaux, Bordeaux, France), Pablo Mir Rivera (Hospital Universitario Virgen del Rocío, Sevilla, Spain), Hidek Mochizuki (Osaka University Hospital, Suita‐shi, Japan), John Morgan (Georgia Regents University, Augusta, GA), Renato Munhoz (Toronto Western Hospital, Toronto, Canada), Noriko Nishikawa (National Center of Neurology and Psychiatry, Kodaira, Japan), John O'Sullivan (Q‐Pharm Pty Limited, Herston, Australia), Tomoko Oeda (National Hospital Organization Utano National Hospital, Kyoto City, Japan), Hideki Oizumi (National Hospital Organization [NHO] Sendai Nishitaga National Hospital, Sendai, Japan), Osamu Onodera (Niigata University Medical & Dental Hospital, Niigata‐shi, Japan), Fabienne Ory‐Magne (Hopital Universitaire Purpan, Toulouse, France), Elizabeth Peckham (Central Texas Neurology Consultants, Round Rock, TX), Ronald Postuma (Montreal Neurological Institute‐Hospital, Montreal, Canada), Aldo Quattrone (Università Magna Graecia di Catanzaro, Catanzaro, Italy), Joseph Quinn (Oregon Health & Science University, Portland, OR), Stefano Ruggieri (IRCCS Istituto Neurologico Mediterraneo Neuromed, Pozzilli, Italy), Justyna Sarna (University of Calgary, Calgary, Canada), Paul E. Schulz (McGovern Medical School, Houston, TX), John Slevin (University of Kentucky Albert B. Chandler Hospital, Lexington, KY), Michele Tagliati (Cedars‐Sinai Medical Center, Beverly Hills, CA), Daryl Wile (Optical Coherence Tomography [OCT] Research University of British Columbia [UBC], Kelowna, Canada), Zbigniew Wszolek (Mayo Clinic, Jacksonville, FL), Tao Xie (University of Chicago, Chicago, IL), Theresa Zesiewicz (University of South Florida, Tampa, FL).

### A.4 Tau Restoration on PSP (TAUROS) MRI Investigators

J.C. Gómez, MD, B. Tijero, MD; R. Villoria, MD (H. de Cruces. Barakaldo, Site Investigator); J. García de Yebenes, MD, J.L. Lopez Sendón, MD (H. Ramón y Cajal. Madrid, Site Investigators); E. Tolosa, MD, M.T. Buongiorno, MD, N. Bargalló, MD (H. Clinic. Barcelona, Site Investigators); J.A. Burguera, MD, I. Martinez, MD (H. La Fe. Valencia, Site Investigators); J. Ruiz‐Martínez, MD, J. Villanua, MD (H. Donostia. San Sebastián, Site Investigator); F. Vivancos, MD, I. Ybot, MD (H. La Paz. Madrid, Site Investigators); M. Aguilar, MD, J.L. Dolz, MD (H. Mutua Terrassa. Terrassa, Site Investigator); M. Boada, MD, A. Lafuente, MD, M.A. Tejero, MD (Fundación ACE: Barcelona, Site Investigators); J.J. López‐Lozano, MD, M. Mata, MD (H. Puerta de Hierro. Madrid, Site Investigators); A. Kupsch, MD, A. Lipp, MD (Virchow‐Klinikum. Berlin, Site Investigators); M. Höllerhage, MD, W.H. Oertel, MD, G. Respondek, MD, M. Stamelou, MD, S. Knake, MD (Universitätsklinikum. Marburg, Site Investigators); D. Berg, MD, W. Maetzler, MD, K.K. Srulijes, MD, A. Gröger, MD (Universitätsklinikum. Tübingen, Site Investigators); A. Ludolph, MD, J. Kassubek, MD (Universitätsklinikum. Ulm, Site Investigators); M. Steiger, MD, K. Tyler, MD (Walton Center. Liverpool, Site Investigators); D.J. Burn, MD, L. Morris, MD (Clinical Ageing Research Unit. Newcastle upon Tyne, Site Investigator); A. Lees, MD, H. Ling, MD, L. Strycharczuk, MD (Reta Lila Weston Institute. London, Site Investigators).

### A.5 The DescribePSP Study Group

Slawek Altenstein (German Center for Neurodegenerative Diseases (DZNE), Berlin, Germany and Neuropsychiatry and Laboratory of Molecular Psychiatry, Department of Psychiatry and Psychotherapy, Charité—Universitätsmedizin Berlin, Berlin, Germany), Mathias Baehr (German Center for Neurodegenerative Diseases [DZNE], Goettingen, Germany, Department of Neurology, University Medical Center, Georg August University, Göttingen, Germany and Cluster of Excellence Nanoscale Microscopy and Molecular Physiology of the Brain [CNMPB], University Medical Center Göttingen, Göttingen, Germany), Miriam Barkhoff (German Center for Neurodegenerative Diseases [DZNE], Bonn, Germany), Claudia Bartels (Department of Psychiatry and Psychotherapy, University Medical Center, University of Goettingen, Germany), Bartl Michael (Department of Neurology, University Medical Center, Georg August University, Göttingen, Germany, and Institute for Neuroimmunology and Multiple Sclerosis Research, University Medical Center Goettingen, Goettingen, Germany), Rudi Beschorner Department of Neuropathology, University of Tübingen, Tübingen, Germany, Center for Neuro‐Oncology, Comprehensive Cancer Center Tübingen‐Stuttgart, University Hospital Tübingen, Germany, Center for Personalized Medicine Tübingen, Eberhard Karls University Tübingen, Germany, and German Cancer Consortium [DKTK], DKFZ partner site Tübingen, Eberhard Karls University of Tübingen, Germany), Aline Beyle (German Center for Neurodegenerative Diseases [DZNE], Bonn, Germany, and Department of Neurology, University of Bonn, Bonn, Germany), Henning Boecker (German Center for Neurodegenerative Diseases [DZNE], Bonn, Germany), Moritz Brandt (German Center for Neurodegenerative Diseases [DZNE], Dresden, Germany, and Department of Neurology, University Hospital Carl Gustav Carus, Technische Universität Dresden, Dresden, Germany), Kathrin Brockmann (German Center for Neurodegenerative Diseases [DZNE], Tübingen, Germany, and Hertie Institute for Clinical Brain Research, Department of Neurodegenerative Diseases, University of Tübingen, Tübingen, Germany), Frederic Brosseron (German Center for Neurodegenerative Diseases [DZNE], Bonn, Germany), Katharina Bürger German Center for Neurodegenerative Diseases [DZNE], Munich, Germany, and Institute for Stroke and Dementia Research [ISD], University Hospital, LMU Munich, Germany), Lena Burow (Department of Psychiatry and Psychotherapy, University Hospital, LMU Munich, Munich, Germany), Cihan Catak (ISD, University Hospital, LMU Munich, Germany), Arda Can Cetindag (Charité—Universitätsmedizin Berlin, corporate member of Freie Universität Berlin and Humboldt‐Universität zu Berlin‐Institute of Psychiatry and Psychotherapy), Nicoleta Carmen Cosma (Charité—Universitätsmedizin Berlin, corporate member of Freie Universität Berlin and Humboldt‐Universität zu Berlin‐Institute of Psychiatry and Psychotherapy), Marcel Daamen (German Center for Neurodegenerative Diseases [DZNE], Bonn, Germany), Eman Dashti (Charité—Universitätsmedizin Berlin, corporate member of Freie Universität Berlin and Humboldt‐Universität zu Berlin‐Institute of Psychiatry and Psychotherapy), Sylvia de Jonge (Department of Psychiatry and Psychotherapy, University Hospital, LMU Munich, Munich, Germany), Peter Dechent (MR‐Research in Neurology and Psychiatry, Georg‐August‐University Göttingen, Germany), Martin Dichgans (German Center for Neurodegenerative Diseases [DZNE], Munich, Germany, and ISD, University Hospital, LMU Munich, Feodor‐Lynen‐Straße 17, 81377 Munich, Germany), Elisabeth Dinter (German Center for Neurodegenerative Diseases [DZNE], Dresden, Germany, and Department of Neurology, University Hospital Carl Gustav Carus, Technische Universität Dresden, Dresden, Germany), Laura Dobisch (German Center for Neurodegenerative Diseases [DZNE], Magdeburg, Germany, and Institute of Cognitive Neurology and Dementia Research, Otto‐von‐Guericke University, Magdeburg, Germany), Angelika Dörr (ISD, University Hospital, LMU Munich, Feodor‐Lynen‐Straße 17, 81377 Munich, Germany), Markus Donix (German Center for Neurodegenerative Diseases [DZNE], Dresden, Germany, and Department of Psychiatry and Psychotherapy, University Hospital Carl Gustav Carus, Technische Universität Dresden, Dresden, Germany), Louisa Droste zu Senden (German Center for Neurodegenerative Diseases [DZNE], Berlin, Germany, Charité Universitätsmedizin Berlin, Department of Psychiatry and Neurosciences, Charité—Universitätsmedizin Berlin, ECRC Experimental and Clinical Research Center), Alexander Drzezga (Department of Nuclear Medicine, Faculty of Medicine and University Hospital Cologne, University of Cologne, Germany, German Center for Neurodegenerative Diseases, Bonn‐Cologne, Germany, and Institute of Neuroscience and Medicine, Molecular Organization of the Brain, Forschungszentrum Jülich, Germany), Emrah Düzel (German Center for Neurodegenerative Diseases [DZNE], Magdeburg, Germany, Institute of Cognitive Neurology and Dementia Research, Otto‐von‐Guericke University, Magdeburg, Germany, and Institute of Cognitive Neuroscience, University College London, London, UK), Martin Dyrba (German Center for Neurodegenerative Diseases [DZNE], Rostock‐Greifswald, Germany), Marie Ehrlich (Charité—Universitätsmedizin Berlin, corporate member of Freie Universität Berlin and Humboldt‐Universität zu Berlin‐Institute of Psychiatry and Psychotherapy), Matthias Endres (German Center for Neurodegenerative Diseases [DZNE], Berlin, Germany, and Department of Neurology with Experimental Neurology, Charité‐Universitätsmedizin Berlin, Berlin, Germany), Tanja Engels (German Center for Neurodegenerative Diseases [DZNE], Bonn, Germany), Ersin Ersözlü (German Center for Neurodegenerative Diseases [DZNE], Berlin, Germany, and Charité—Universitätsmedizin Berlin, corporate member of Freie Universität Berlin and Humboldt‐Universität zu Berlin‐Institute of Psychiatry and Psychotherapy), Michael Ewers (German Center for Neurodegenerative Diseases [DZNE], Munich, Germany, and ISD, University Hospital, LMU Munich, Germany), Jennifer Faber (German Center for Neurodegenerative Diseases [DZNE], Bonn, Germany, and Department of Neurology, University of Bonn, Bonn, Germany), Björn Falkenburger (German Center for Neurodegenerative Diseases [DZNE], Dresden, Germany, and Department of Neurology, University Hospital Carl Gustav Carus, Technische Universität Dresden, Dresden, Germany), Frederike Fenski (Charité—Universitätsmedizin Berlin, corporate member of Freie Universität Berlin and Humboldt‐Universität zu Berlin‐Institute of Psychiatry and Psychotherapy), Klaus Fließbach (German Center for Neurodegenerative Diseases [DZNE], Bonn, Germany, and Department of Old Age Psychiatry and Cognitive Disorders, University Hospital Bonn and University of Bonn, Bonn, Germany), Agnes Flöel (Department of Neurology, University Medicine Greifswald, 17475 Greifswald, Germany, and German Centre for Neurodegenerative Diseases, Rostock and Greifswald, Germany), Silka Dawn Freiesleben (Charité—Universitätsmedizin Berlin, corporate member of Freie Universität Berlin and Humboldt‐Universität zu Berlin‐Institute of Psychiatry and Psychotherapy), Franca Laura Fries (German Center for Neurodegenerative Diseases [DZNE], Tübingen, Germany, Hertie Institute for Clinical Brain Research, Department of Neurodegenerative Diseases, University of Tübingen, Tübingen, Germany), Ingo Frommann (German Center for Neurodegenerative Diseases [DZNE], Bonn, Germany), Thomas Gasser (German Center for Neurodegenerative Diseases [DZNE], Tübingen, Germany, Hertie Institute for Clinical Brain Research, Department of Neurodegenerative Diseases, University of Tübingen, Tübingen, Germany), Wenzel Glanz (German Center for Neurodegenerative Diseases [DZNE], Magdeburg, Germany, Institute of Cognitive Neurology and Dementia Research, Otto‐von‐Guericke University, Magdeburg, Germany, and Clinic for Neurology, Medical Faculty, University Hospital Magdeburg, 39120 Magdeburg, Germany), Doreen Goerss (Clinic for Neurology, Medical Faculty, University Hospital Magdeburg, 39120 Magdeburg, Germany), Doreen Grieger‐Klose (German Center for Neurodegenerative Diseases [DZNE], Magdeburg, Germany), Marcus Grobe‐Einsler (German Center for Neurodegenerative Diseases [DZNE], Bonn, Germany, and Department of Neurology, University Hospital Bonn, Bonn, Germany), Martin Grond (Kreisklinikum Siegen GmbH, Department of Neurology, Siegen, Germany), Selim Üstün Gürsel (German Center for Neurodegenerative Diseases [DZNE], Munich, Germany, and Department of Psychiatry and Psychotherapy, University Hospital, LMU Munich, Munich, Germany), Annett Halle (German Center for Neurodegenerative Diseases [DZNE], Bonn, Germany), Niels Hansen (Department of Psychiatry and Psychotherapy, University Medical Center Goettingen, University of Goettingen, Germany), Deike Hartmann (German Center for Neurodegenerative Diseases [DZNE], Magdeburg, Germany), Lina Hassoun (Department of Psychiatry and Psychotherapy, University Medical Center Goettingen, University of Goettingen, Germany), Robert Haussmann (Department of Psychiatry and Psychotherapy, University Hospital Carl Gustav Carus, Technische Universität Dresden, Dresden, Germany), Julian Hellmann‐Regen (German Center for Neurodegenerative Diseases [DZNE], Berlin, Germany, Charité Universitätsmedizin Berlin, Department of Psychiatry and Neurosciences, and Charité—Universitätsmedizin Berlin, ECRC Experimental and Clinical Research Center), Guido Hennes (German Center for Neurodegenerative Diseases [DZNE], Bonn, Germany), Wiebke Hermann (German Center for Neurodegenerative Diseases [DZNE], Rostock‐Greifswald, Germany, and Department of Neurology, University Medical Centre, Rostock, Germany), Andreas Hermann (German Center for Neurodegenerative Diseases [DZNE], Rostock‐Greifswald, Germany, and Department of Neurology, University Medical Centre, Rostock, Germany), Jochen Herms (German Center for Neurodegenerative Diseases [DZNE], Munich, Germany, Munich Cluster for Systems Neurology (SyNergy) Munich, Munich, Germany, and Department of Neurology, School of Medicine, University Hospital München rechts der Isar, Technical University of Munich, Munich, Germany), Gabi Herrmann (German Center for Neurodegenerative Diseases [DZNE], Bonn, Germany), Stefan Hetzer (Berlin Center for Advanced Neuroimaging, Charité—Universitätsmedizin Berlin, Berlin, Germany), Petra Hinderer (German Center for Neurodegenerative Diseases [DZNE], Tübingen, Germany), Günther U. Höglinger (ISD, University Hospital, LMU Munich, Feodor‐Lynen‐Straße 17, 81377 Munich, Germany, and Department of Neurology, University Hospital of Munich, LMU Munich, Munich, Germany), Ildiko Horvath (German Center for Neurodegenerative Diseases [DZNE], Munich, Germany), Nicole Hujer (Charité—Universitätsmedizin Berlin, corporate member of Freie Universität Berlin and Humboldt‐Universität zu Berlin‐Institute of Psychiatry and Psychotherapy), Enise Irem Incesoy (German Center for Neurodegenerative Diseases [DZNE], Magdeburg, Germany, and Institute of Cognitive Neurology and Dementia Research, Otto‐von‐Guericke University, Magdeburg, Germany), Alexander Jäck (ISD, University Hospital, LMU Munich, Feodor‐Lynen‐Straße 17, 81377 Munich, Germany, and Department of Neurology, University Hospital of Munich, LMU Munich, Munich, Germany), Daniel Janowitz (German Center for Neurodegenerative Diseases [DZNE], Munich, Germany, and ISD, University Hospital, LMU Munich, Feodor‐Lynen‐Straße 17, 81377 Munich, Germany), Frank Jessen (German Center for Neurodegenerative Diseases [DZNE], Bonn, Germany, Excellence Cluster on Cellular Stress Responses in Aging‐Associated Diseases [CECAD], University of Cologne, Germany, and Department of Psychiatry and Psychotherapy, University of Cologne, Medical Faculty, Cologne, Germany), Lorraine Jost (German Center for Neurodegenerative Diseases [DZNE], Bonn, Germany), Pascal Kalbhen (Department of Neurology, University of Bonn, Bonn, Germany), Jan Kassubek (Department of Neurology, University of Ulm, Ulm, Germany, and German Center for Neurodegenerative Diseases [DZNE], Ulm, Germany), Sabrina Katzdobler (ISD, University Hospital, LMU Munich, Feodor‐Lynen‐Straße 17, 81377 Munich, Germany, and Department of Neurology, University Hospital of Munich, LMU Munich, Munich, Germany), Ingo Kilimann (German Center for Neurodegenerative Diseases [DZNE], Rostock‐Greifswald, Germany, and Department of Psychosomatic Medicine, Rostock University Medical Center, Rostock, Germany), Okka Kimmich (German Center for Neurodegenerative Diseases [DZNE], Bonn, Germany, and Department of Neurology, University of Bonn, Bonn, Germany), Luca Kleinadam (German Center for Neurodegenerative Diseases [DZNE], Bonn, Germany, and Department of Old Age Psychiatry and Cognitive Disorders, University Hospital Bonn and University of Bonn, Bonn, Germany), Thomas Klockgether (German Center for Neurodegenerative Diseases [DZNE], Bonn, Germany, and Department of Neurology, University of Bonn, Bonn, Germany), Xenia Kobeleva (German Center for Neurodegenerative Diseases [DZNE], Bonn, Germany, and Department of Neurology, University of Bonn, Bonn, Germany), Patricia Krause (Movement Disorder and Neuromodulation Unit, Department of Neurology, Charité, University Medicine Berlin, Charité—Berlin, Germany), Elke Kuder‐Buletta (German Center for Neurodegenerative Diseases [DZNE], Tübingen, Germany), Andrea Kühn (German Center for Neurodegenerative Diseases [DZNE], Berlin, Germany, and Movement Disorder and Neuromodulation Unit, Department of Neurology, Charité, University Medicine Berlin, Charité—Berlin, Germany), Carolin Kurz (Munich Cluster for Systems Neurology [SyNergy] Munich, Munich, Germany), Catharina lange (Department of Nuclear Medicine, Charité—Universitätsmedizin Berlin, corporate member of Freie Universität Berlin, and Humboldt‐Universität zu Berlin, Berlin, Germany), Chris Lappe (Department of Neurology, University Medical Centre, Rostock, Germany), Christoph Laske (German Center for Neurodegenerative Diseases [DZNE], Tübingen, Germany, Section for Dementia Research, Department of Psychiatry and Psychotherapy, Hertie Institute for Clinical Brain Research, University of Tübingen, Tübingen, Germany), Johannes Levin (German Center for Neurodegenerative Diseases [DZNE], Munich, Germany, Munich Cluster for Systems Neurology [SyNergy] Munich, Munich, Germany, and Department of Neurology, University Hospital of Munich, LMU Munich, Munich, Germany), Sandra Lichte Schneider (Kreisklinikum Siegen GmbH, Department of Neurology, Siegen, Germany), Paul Lingor (German Center for Neurodegenerative Diseases [DZNE], Munich, Germany, Munich Cluster for Systems Neurology [SyNergy] Munich, Munich, Germany, and Department of Neurology, and Department of Neurology, School of Medicine, University Hospital München rechts der Isar, Technical University of Munich, Munich, Germany), Matthias Löhle (German Center for Neurodegenerative Diseases [DZNE], Rostock‐Greifswald, Germany, and Department of Neurology, University Medical Centre, Rostock, Germany), Albert C. Ludolph (Department of Neurology, University of Ulm, Ulm, Germany, and German Center for Neurodegenerative Diseases [DZNE], Ulm, Germany), Falk Lüsebrink (German Center for Neurodegenerative Diseases [DZNE], Magdeburg, Germany), Cornelia McCormick (German Center for Neurodegenerative Diseases [DZNE], Bonn, Germany), David Mengel (Division of Translational Genomics of Neurodegenerative Diseases, Hertie Institute for Clinical Brain Research and Center of Neurology, University of Tübingen, Tübingen, Germany), Coraline Metzger (German Center for Neurodegenerative Diseases [DZNE], Magdeburg, Germany), Carolin Miklitz (German Center for Neurodegenerative Diseases [DZNE], Bonn, Germany, and Department of Old Age Psychiatry and Cognitive Disorders, University Hospital Bonn and University of Bonn, Bonn, Germany), Matthias Munk (German Center for Neurodegenerative Diseases [DZNE], Tübingen, Germany and Department of Psychiatry and Psychotherapy, University of Tübingen, Tübingen, Germany), Manuela Neumann (Department of Psychiatry and Psychotherapy, University of Tübingen, Tübingen, Germany, and Department of Neuropathology, University of Tübingen, Tübingen, Germany), Demet Oender (German Center for Neurodegenerative Diseases [DZNE], Bonn, Germany), Robert Perneczky (German Center for Neurodegenerative Diseases [DZNE], Munich, Germany, Department of Psychiatry and Psychotherapy, University Hospital, LMU Munich, Munich, Germany, Munich Cluster for Systems Neurology [SyNergy] Munich, Munich, Germany, and Ageing Epidemiology Research Unit, School of Public Health, Imperial College London, London, United Kingdom), Oliver Peters (German Center for Neurodegenerative Diseases [DZNE], Berlin, Germany, and Charité—Universitätsmedizin Berlin, corporate member of Freie Universität Berlin and Humboldt‐Universität zu Berlin‐Institute of Psychiatry and Psychotherapy), Gabor C. Petzold (German Center for Neurodegenerative Diseases [DZNE], Bonn, Germany, and German Center for Neurodegenerative Diseases [DZNE], Berlin, Germany), Henrike Pfaff (Department of Neurology, University Medical Centre, Rostock, Germany), Alexandra Polcher (German Center for Neurodegenerative Diseases [DZNE], Bonn, Germany), Lukas Preis (Charité—Universitätsmedizin Berlin, corporate member of Freie Universität Berlin and Humboldt‐Universität zu Berlin‐Institute of Psychiatry and Psychotherapy), Josef Priller (German Center for Neurodegenerative Diseases [DZNE], Berlin, Germany, Neuropsychiatry and Laboratory of Molecular Psychiatry, Department of Psychiatry and Psychotherapy, Charité—Universitätsmedizin Berlin, Berlin, Germany, Department of Psychiatry and Psychotherapy, School of Medicine and Health, Technical University of Munich, and German Center for Mental Health (DZPG), Munich, Germany, and University of Edinburgh and UK DRI, Edinburgh, UK), Johannes Prudlo (German Center for Neurodegenerative Diseases [DZNE], Rostock‐Greifswald, Germany, and Department of Neurology, University Medical Centre, Rostock, Germany), Dominik Pürner (Department of Neurology, School of Medicine, University Hospital München rechts der Isar, Technical University of Munich, Munich, Germany), Veronika Purrer (German Center for Neurodegenerative Diseases [DZNE], Bonn, Germany, and Department of Neurology, University of Bonn, Bonn, Germany), Boris Rauchmann (Department of Psychiatry and Psychotherapy, University Hospital, LMU Munich, Munich, Germany, Sheffield Institute for Translational Neuroscience [SITraN], University of Sheffield, Sheffield, UK, and Department of Neurology, University Hospital of Munich, LMU Munich, Munich, Germany), Alfredo Ramirez (German Center for Neurodegenerative Diseases [DZNE], Bonn, Germany, Department of Old Age Psychiatry and Cognitive Disorders, University Hospital Bonn and University of Bonn, Bonn, Germany, Excellence Cluster on Cellular Stress Responses in Aging‐Associated Diseases (CECAD), University of Cologne, Germany, Division of Neurogenetics and Molecular Psychiatry, Department of Psychiatry, University of Cologne, Medical Faculty, Cologne, Germany, and Department of Psychiatry and Glenn Biggs Institute for Alzheimer's and Neurodegenerative Diseases, San Antonio, TX, USA), Heike Raum (German Center for Neurodegenerative Diseases [DZNE], Rostock‐Greifswald, Germany), Sandra Röske (German Center for Neurodegenerative Diseases [DZNE], Bonn, Germany), Christin Ruß (German Center for Neurodegenerative Diseases [DZNE], Bonn, Germany), Petr Sabik (German Center for Neurodegenerative Diseases [DZNE], Rostock‐Greifswald, Germany), Yilmaz Sagik (German Center for Neurodegenerative Diseases [DZNE], Bonn, Germany), Klaus Scheffler (Department for Biomedical Magnetic Resonance, University of Tübingen, Tübingen, Germany), Monika Schmidt (German Center for Neurodegenerative Diseases [DZNE], Rostock‐Greifswald, Germany), Anja Schneider (German Center for Neurodegenerative Diseases [DZNE], Bonn, Germany, and Department of Old Age Psychiatry and Cognitive Disorders, University Hospital Bonn and University of Bonn, Bonn, Germany), Luisa‐Sophie Schneider (Charité—Universitätsmedizin Berlin, corporate member of Freie Universität Berlin and Humboldt‐Universität zu Berlin‐Institute of Psychiatry and Psychotherapy), Ludger Schoels (German Center for Neurodegenerative Diseases [DZNE], Tübingen, Germany, and Hertie Institute for Clinical Brain Research, Department of Neurodegenerative Diseases, University of Tübingen, Tübingen, Germany), Björn Schott (German Center for Neurodegenerative Diseases [DZNE], Goettingen, Germany, Department of Psychiatry and Psychotherapy, University Medical Center Goettingen, University of Goettingen, Germany, and Leibniz Institute for Neurobiology, Brenneckestr. 6, 39118 Magdeburg, Germany), Heike Schulz (German Center for Neurodegenerative Diseases [DZNE], Rostock‐Greifswald, Germany), Franziska Schulze (Institute of Cognitive Neurology and Dementia Research, Otto‐von‐Guericke University, Magdeburg, Germany), Sebastian Sodenkamp (German Center for Neurodegenerative Diseases [DZNE], Tübingen, Germany, and Department of Psychiatry and Psychotherapy, University of Tübingen, Tübingen, Germany), Annika Spottke (German Center for Neurodegenerative Diseases [DZNE], Bonn, Germany, and Department of Neurology, University of Bonn, Bonn, Germany), Eike Spruth (German Center for Neurodegenerative Diseases [DZNE], Berlin, Germany, and Neuropsychiatry and Laboratory of Molecular Psychiatry, Department of Psychiatry and Psychotherapy, Charité—Universitätsmedizin Berlin, Berlin, Germany), Melina Stark (German Center for Neurodegenerative Diseases [DZNE], Bonn, Germany, and Department of Old Age Psychiatry and Cognitive Disorders, University Hospital Bonn and University of Bonn, Bonn, Germany), Matthis Synofzik (German Center for Neurodegenerative Diseases [DZNE], Tübingen, Germany, and Division of Translational Genomics of Neurodegenerative Diseases, Hertie Institute for Clinical Brain Research and Center of Neurology, University of Tübingen, Tübingen, Germany), Patricia Sulzer (German Center for Neurodegenerative Diseases [DZNE], Tübingen, Germany), Stefan Teipel (German Center for Neurodegenerative Diseases [DZNE], Rostock‐Greifswald, Germany, and Department of Psychosomatic Medicine, Rostock University Medical Center, Rostock, Germany), Christoph van Riesen (German Center for Neurodegenerative Diseases [DZNE], Goettingen, Germany, and Department of Neurology, University Medical Center, Georg August University, Göttingen, Germany), Ina R. Vogt (German Center for Neurodegenerative Diseases [DZNE], Bonn, Germany), Michael Wagner (German Center for Neurodegenerative Diseases [DZNE], Bonn, Germany, and Department of Old Age Psychiatry and Cognitive Disorders, University Hospital Bonn and University of Bonn, Bonn, Germany), Endy Weidinger (German Center for Neurodegenerative Diseases [DZNE], Munich, Germany, and Department of Neurology, University Hospital of Munich, LMU Munich, Munich, Germany), Carlo Wilke (Division of Translational Genomics of Neurodegenerative Diseases, Hertie Institute for Clinical Brain Research and Center of Neurology, University of Tübingen, Tübingen, Germany), Jens Wiltfang (German Center for Neurodegenerative Diseases [DZNE], Goettingen, Germany, Department of Psychiatry and Psychotherapy, University Medical Center Goettingen, University of Goettingen, Germany, and Neurosciences and Signaling Group, Institute of Biomedicine [iBiMED], Department of Medical Sciences, University of Aveiro, Aveiro, Portugal), Steffen Wolfsgruber (German Center for Neurodegenerative Diseases [DZNE], Bonn, Germany, and Department of Old Age Psychiatry and Cognitive Disorders, University Hospital Bonn and University of Bonn, Bonn, Germany), Ullrich Wüllner (German Center for Neurodegenerative Diseases [DZNE], Bonn, Germany, and Department of Old Age Psychiatry and Cognitive Disorders, University Hospital Bonn and University of Bonn, Bonn, Germany), Renat Yakupov (German Center for Neurodegenerative Diseases [DZNE], Magdeburg, Germany), Heike Zech (German Center for Neurodegenerative Diseases [DZNE], Goettingen, Germany, and Department of Psychiatry and Psychotherapy, University Medical Center Goettingen, University of Goettingen, Germany), Inga Zerr (German Center for Neurodegenerative Diseases [DZNE], Goettingen, Germany, and Department of Neurology, University Medical Center, Georg August University, Göttingen, Germany), Gabriel Ziegler (German Center for Neurodegenerative Diseases [DZNE], Magdeburg, Germany), Adelgunde Zollver (ISD, University Hospital, LMU Munich, Germany).

## Appendix B Alzheimer's Disease Neuroimaging Initiative (ADNI)

A complete list of ADNI investigators can be found at: http://adni.loni.usc.edu/wp-content/uploads/how_to_apply/ADNI_Acknowledgement_List.pdf.
