# Supplementary figures and images for: Brain Atrophy Does Not Predict Clinical Progression in Progressive Supranuclear Palsy
Source: Mov Disord. 2025 Aug 30;40(11):2517–30. doi: 10.1002/mds.70026 (PMC12661634; doi:10.1002/mds.70026)

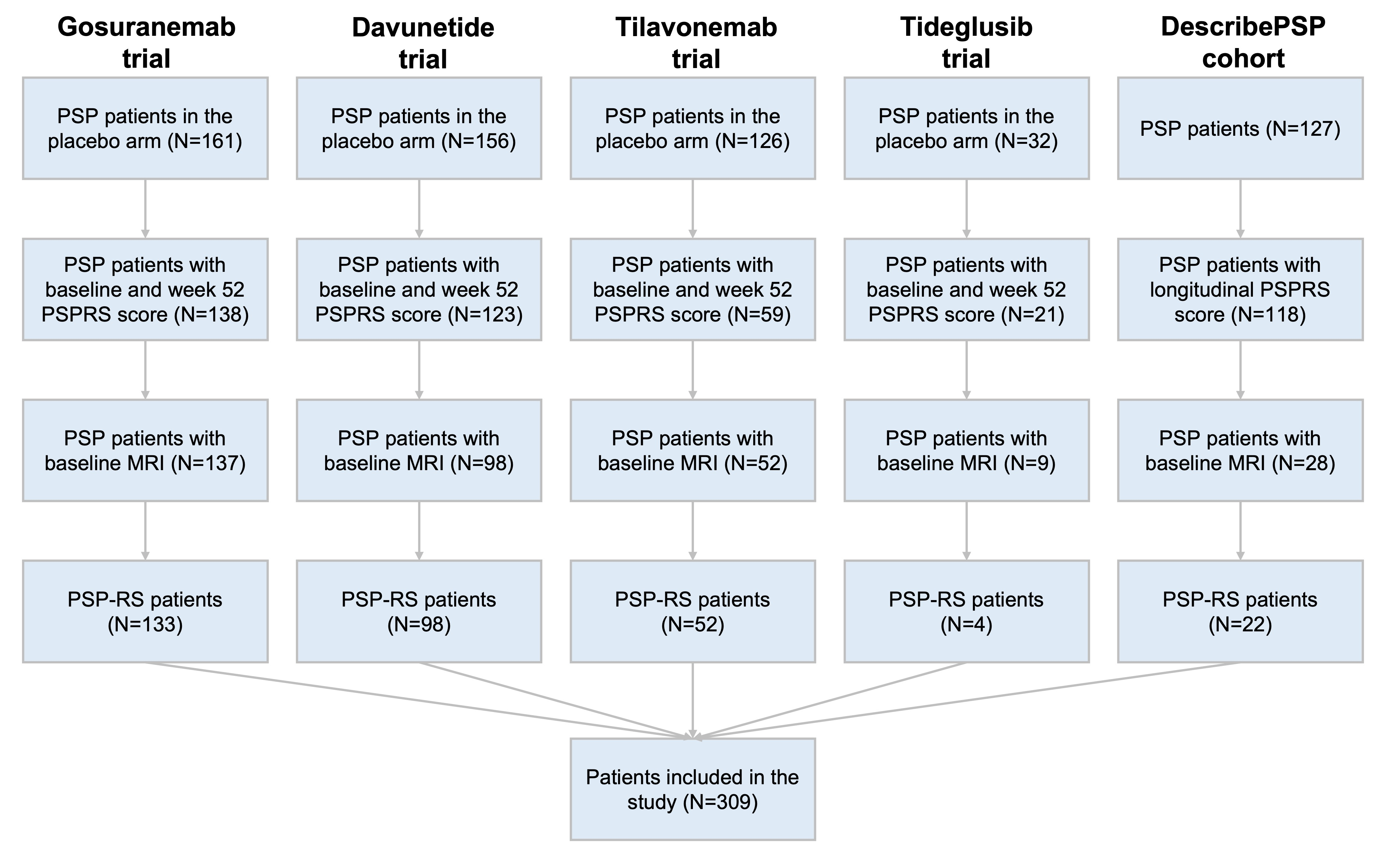

Supplement: Supplementary file 1 — Supplementary Figure S1. A flowchart showing the study inclusion/exclusion procedures. Progressive supranuclear palsy–Richardson's syndrome (PSP‐RS) patients with available longitudinal clinical data, including the PSP rating scale score and available baseline brain magnetic resonance imaging (MRI), were selected from the placebo arms of clinical trials and from the DescribePSP cohort observational study. The final patient cohort included 309 PSP‐RS patients. [file MDS-40-2517-s004.tif]

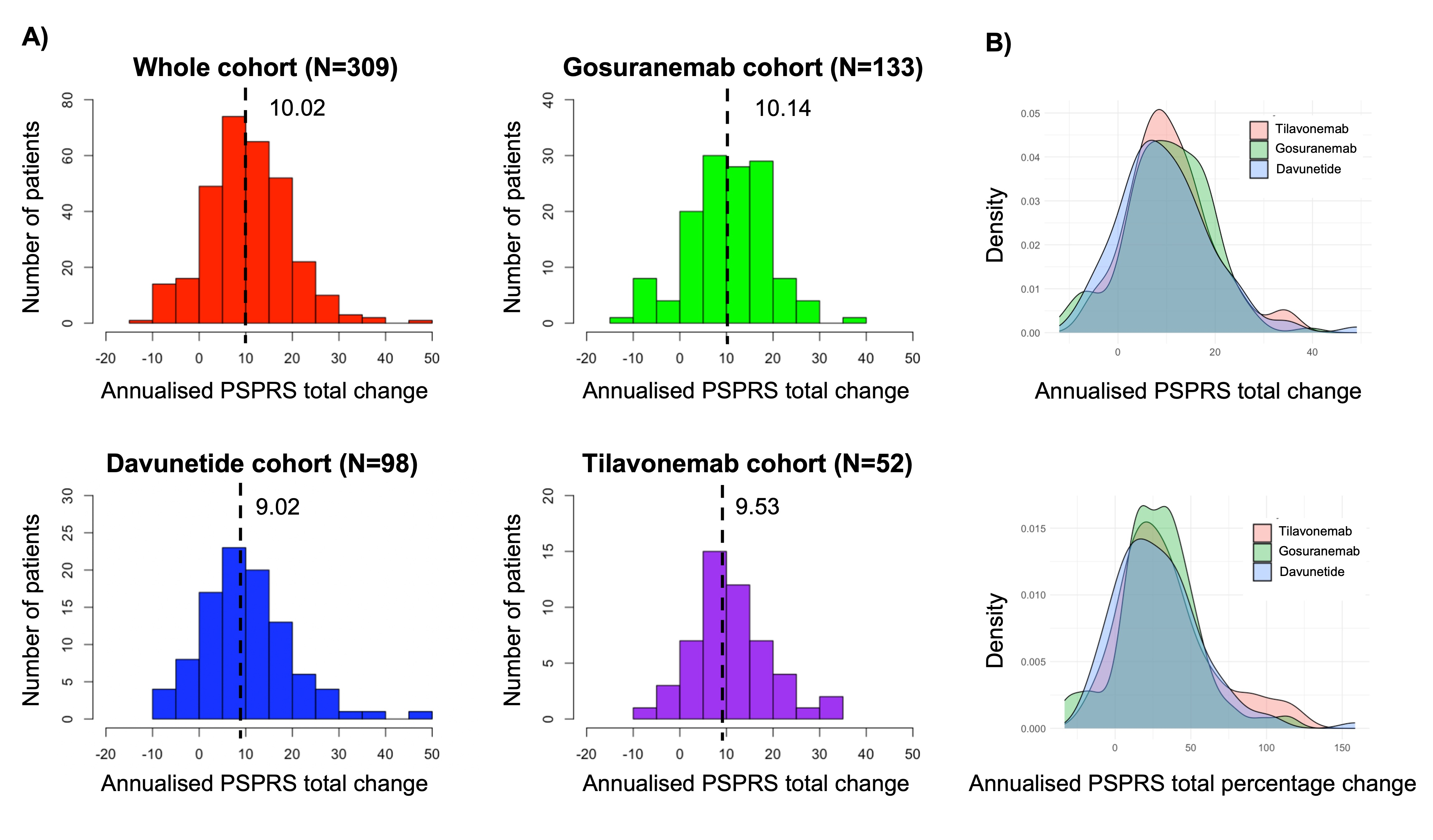

Supplement: Supplementary file 2 — Supplementary Figure S2. (A) The left part of the figure includes histograms showing the annualized PSPRS total score absolute change in PSP‐RS patients in the whole study cohort (red) and in the largest subcohorts from different trials (green, blue, and violet). In each plot, the black dotted line represents the median value, which is also shown in B. The right part of the figure includes density plots of both percentage and absolute PSPRS total score change value distributions, allowing easy visual comparison of clinical progression rates across cohorts. PSP‐RS, progressive supranuclear palsy–Richardson's syndrome; PSPRS, PSP rating scale. [file MDS-40-2517-s007.tif]

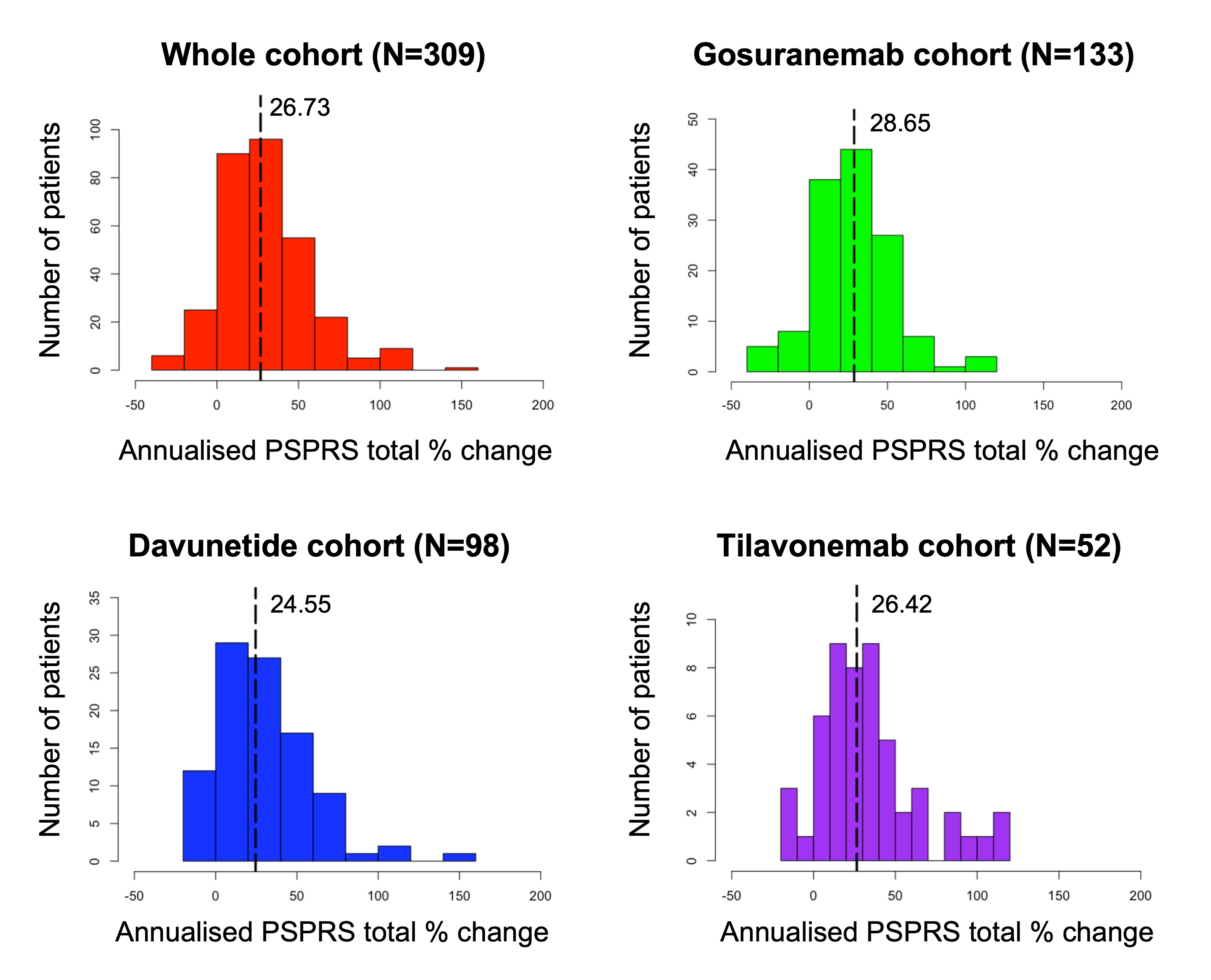

Supplement: Supplementary file 3 — Supplementary Figure S3. Histograms showing the annualized PSPRS total score percentage change in PSP‐RS patients in the whole study cohort (red) and in the largest subcohorts from different trials (green, blue, and violet). In each plot, the black dotted line represents the median value, which is also shown in the figure. PSP‐RS, progressive supranuclear palsy–Richardson's syndrome; PSPRS, PSP rating scale. [file MDS-40-2517-s001.tif]

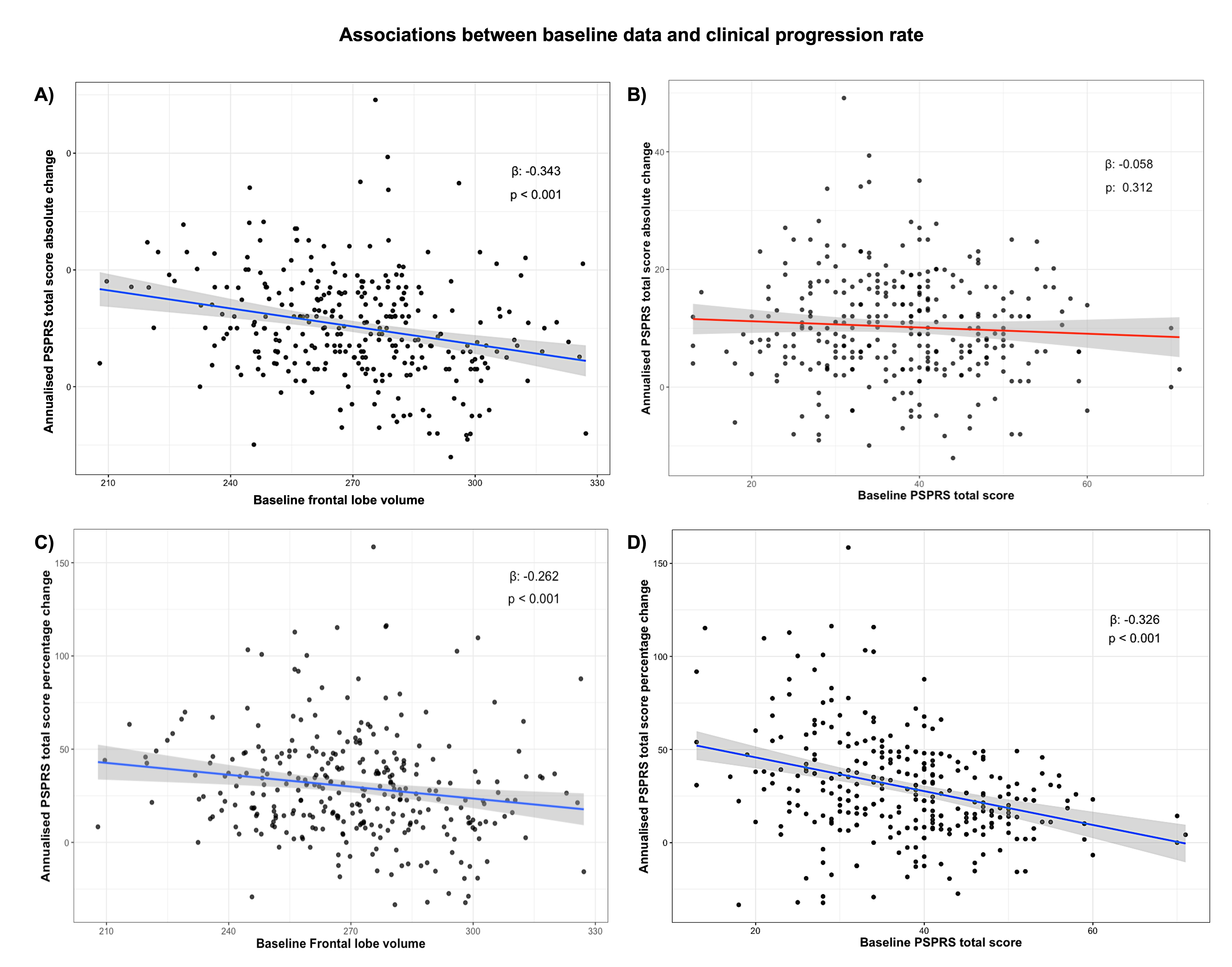

Supplement: Supplementary file 4 — Supplementary Figure S4. Scatterplots showing the strongest associations of baseline clinical and imaging features with longitudinal clinical progression as measured by PSP rating scale (PSPRS). On the left, the associations between baseline frontal lobe volume (in mL) and the annualized longitudinal PSPRS total absolute (A) or percentage score change (C); on the right, the association between baseline PSPRS total score and the annualized longitudinal PSPRS total score absolute (B) or percentage change (D). The blue color was used for significant associations, and the red color for nonsignificant associations. [file MDS-40-2517-s009.tif]

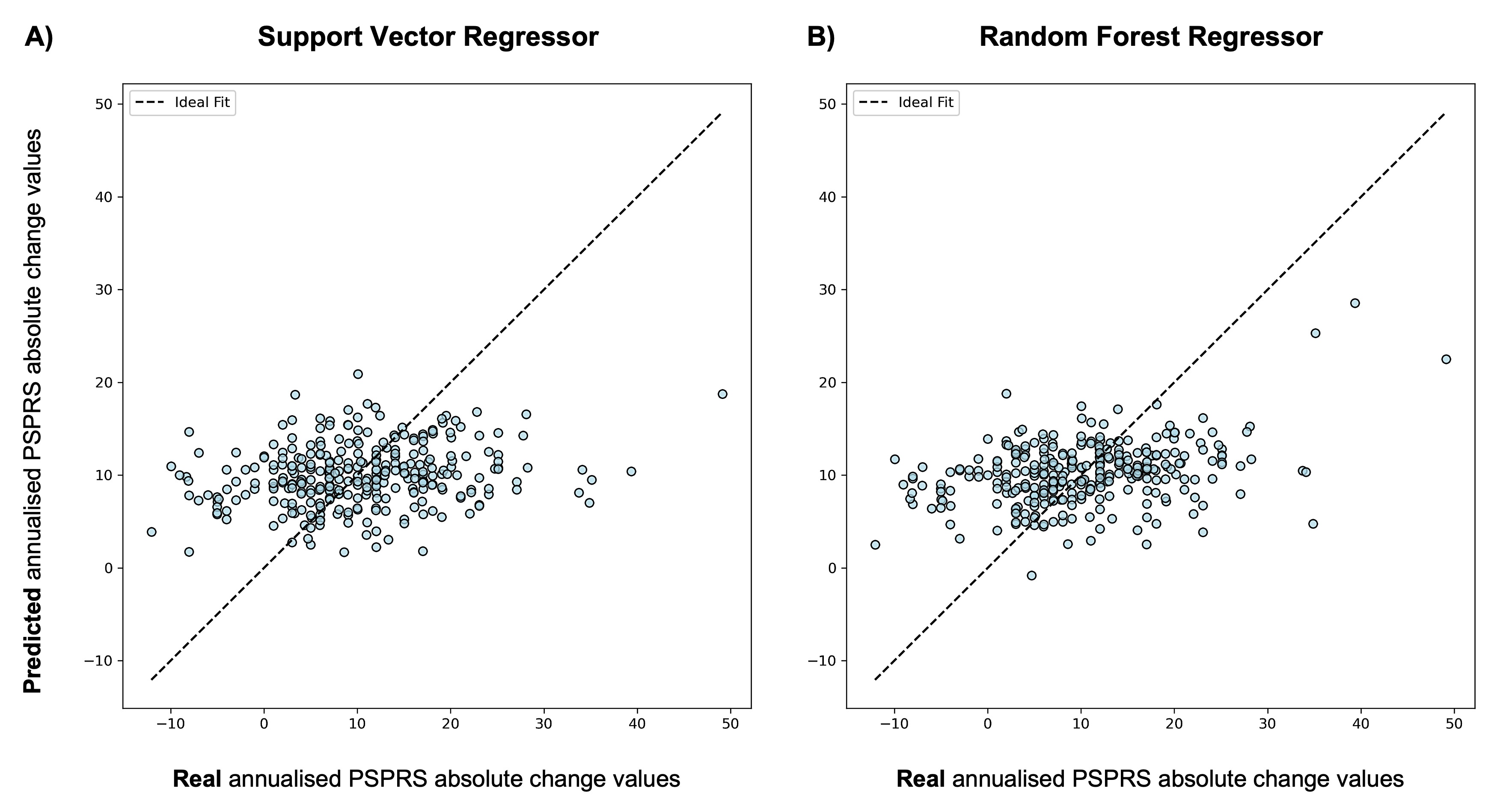

Supplement: Supplementary file 5 — Supplementary Figure S5. Scatterplots showing on x‐axis the real annualized PSP‐RS total score absolute change, and on the y‐axis the values predicted by support vector regression (A) or random forest regression (B) models in the validation folds using baseline clinical and volumetric data. Black dotted lines show the ideal fit. [file MDS-40-2517-s003.tif]
