# Supplementary material for: Brain Atrophy Does Not Predict Clinical Progression in Progressive Supranuclear Palsy
Source: Mov Disord. 2025 Aug 30;40(11):2517–30. doi: 10.1002/mds.70026 (PMC12661634; doi:10.1002/mds.70026)
Supplement: Supplementary file 6 — Data S1. Supporting information. [file MDS-40-2517-s005.docx]

**Supplementary materials**

**Study participants**

This study included PSP-RS patients from the placebo arms of four recent randomized controlled trials (NCT03068468,^1^ NCT01110720,^2^ NCT02985879,^3^ NCT01049399)^4^ which studied patients according to similar inclusion–exclusion criteria and protocols for 52 weeks, and from the DescribePSP network cohort,^5^ which is large German multicentre research network on PSP prospectively collecting clinical and imaging patient data. Ethics approval was obtained at each site from the local ethics committee, and all participants provided written informed consent.

**Gosuranemab Trial (NCT03068468)**

In the NCT03068468 trial, PSP patients were recruited at 90 centres across 13 countries (Australia, Austria, Canada, Germany, Spain, France, the United Kingdom, Greece, Italy, Japan, South Korea, Russia and the United States), between April 2017 and September 2019.^1^ Participants were assigned to receive either gosuranemab (formerly BMS-986168 / IPN007 / BIIB092) or placebo for 52 weeks. Relevant inclusion criteria were: (1) age between 41-86 years and body weight 43–120 kg; (2) history of postural instability or falls during the first 3 years from disease onset, vertical supranuclear gaze palsy or slow velocity of vertical saccades, and an akinetic-rigid syndrome; (3) have PSP symptoms for less than 5 years; (4) be able to ambulate independently or with limited assistance; (5) have a mini-mental state examination (MMSE) score of at least 20; (6) live outside a nursing home or dementia care facility; (7) no other notable neurological or psychiatric disorders including Alzheimer’s disease, dementia with Lewy bodies, prion disease, Parkinson’s disease, hydrocephalus or clinically relevant cerebrovascular disease. MRI data was collected on 1.5T or 3T scanners with magnetization prepared 3D T1-weighted sequences 1.0 x 1.0 x 1.2 mm isotropic voxels acquired following the Alzheimer’s Disease Neuroimaging Initiative (https://adni.loni.usc.edu) recommendations for volumetric analysis.

### Davunetide (AL-108-231) Trial (NCT01110720)

In the NCT01110720 trial, PSP patients were recruited at 48 centres across 6 countries (Australia, Canada, France, Germany, the United Kingdom, and the United States), between September 2010 and November 2012.^2^ Participants were randomly assigned in a 1:1 ratio to davunetide or placebo for 52 weeks. Relevant inclusion criteria were: (1) age at disease onset between 41-85 years; (2) at least a 12-month history of postural instability or falls during the first 3 years from disease onset, supranuclear ophthalmoplegia or reduced downward saccade velocity, and prominent axial rigidity; (3) have PSP symptoms for either less than 5 years, or more than 5 years with a PSPRS score or no more than 40; (4) be able to ambulate independently (or walk 5 steps with minimal assistance); (5) have a mini-mental state examination (MMSE) score of at least 15; (6) live outside a nursing home or dementia care facility. MRI data was collected on 1.5 or 3T scanners with magnetization prepared 3D T1-weighted sequences 1.0 x 1.0 x 1.0 mm isotropic voxels acquired following the Alzheimer’s Disease Neuroimaging Initiative (https://adni.loni.usc.edu) recommendations for volumetric analysis.

**Tilavonemab (ABBV-8E12) Trial (NCT02985879)**

In the NCT02985879 trial, PSP patients were recruited at 66 centres across 8 countries (Australia, Canada, France, Germany, Italy, Japan, Spain, and the USA), between December 2016 and December 2018.^3^ Participants were randomly assigned in a 1:1:1 ratio to tilavonemab 2000 mg, tilavonemab 4000 mg, or placebo for 52 weeks. Relevant inclusion criteria were: (1) age at disease onset ≥ 40 years; (2) presence of vertical supranuclear gaze palsy or slowing of vertical saccades coupled postural instability with falls in the first 3 years of symptoms (3) have PSP symptoms for either less than 5 years (4) be able to ambulate independently (or walk 5 steps with minimal assistance); (5) reliable study partner (caregiver, family member, social worker, or friend).

**Tideglusib Trial (NCT01049399)**

In the NCT01049399 trial, PSP patients were recruited at 24 centres across 4 countries (Germany, Spain, the United Kingdom and the United States), between December 2009 and November 2011.^4^ Participants were randomly assigned to tideglusib 600 mg, tideglusib 800 mg or placebo with a 2:2:1 ratio for 52 weeks. Relevant inclusion criteria were: (1) age at disease onset between 40 and 85 years; (2) possible or probable National Institute of Neurological Disorders and Stroke and Society for PSP (NINDS- SPSP) criteria (3) Mild-to-moderate stage of disease severity according to score of 1 to 4 in Golbe Staging System; (4) Brain magnetic resonance imaging (MRI) study within 24 months before baseline visit excluding other potential causes of parkinsonism, especially cerebrovascular lesions and space occupying lesions. MRI data was collected on 1.5 or 3T scanners with magnetization prepared 3D T1-weighted sequences 1.0 x 1.0 x 1.0 mm isotropic voxels acquired following the Alzheimer’s Disease Neuroimaging Initiative (https://adni.loni.usc.edu) recommendations for volumetric analysis.

**DescribePSP Network cohort**

DescribePSP (DZNE Clinical Register Study of Neurodegenerative Disorders - PSP) is a large German multicentre research network set up in 2015 organized by the German Centre for Neurodegenerative Diseases (DZNE), prospectively collecting comprehensive clinical data, imaging data and biomaterials of patients with PSP.^5^ Patients with a clinical diagnosis of PSP according to the MDS diagnostic criteria^1^ were consecutively enrolled in the observational DescribePSP study at 11 tertiary care centres with expertise in movement disorders, in Berlin, Bonn, Dresden, Gottingen, Greifswald, Hanover, Cologne, Magdeburg, Munich, Rostock, and Tubingen. Brain MRI data was collected on 3T scanners acquired following the Alzheimer’s Disease Neuroimaging Initiative (https://adni.loni.usc.edu) recommendations for volumetric analysis.

**Healthy control group**

This multicohort group included healthy controls (HC) aged above 45 years old, was described in a previous publication^6^ and included individuals from the Alzheimer’s-Disease-Neuroimaging-Initiative (ADNI, n=116), the Degeneration-Controls-and-Relatives (DANCER) cohort (n=70) and a previously published multicentre German HC cohort (n=72).^7^

The 116 HC were selected from ADNI2 and ADNI3, based on normal 18F-florbetapir or 18F-florbetaben amyloid-PET (using pre-established Aβ positivity standardized uptake value ratio threshold of 1.11 for 18F-florbetapir and 1.08 for 18F-florbetaben amyloid-PET) and available baseline and 52-week-follow-up longitudinal brain 3D T1-weighted 3T-MRI. The DANCER (Degeneration Controls and Relatives) HC cohort is part of the same large DZNE network of DescribePSP which includes parallel cohorts of several neurodegenerative diseases and HC. Participants included in the DANCER cohort were recruited at the same centres over the same period of time, and underwent brain 3T MRI with the same acquisition protocol mentioned in the DescribePSP network paragraph. in 82 individuals; subject aged >45 years with available brain MRI (n=70) were included in this study. The remaining 73 HC were recruited between 2009 and 2013 at five German university hospitals (Marburg, Düsseldorf, Frankfurt, Freiburg, and Ulm).^7^ The brain MRI protocol comprised sagittal T1-weighted 3D magnetization prepared rapid gradient echo (MPRAGE) sequences (1.0 x 1.0 x 1.0 mm^3^ resolution), T2-weighted axial and sagittal images, and axial fluid-attenuated inversion recovery (FLAIR) images. Brain MRI data was collected on 1.5 or 3T scanners.

**MRI processing**

The 3D T1-weighted MR images were pseudonymized and processed through an automated pipeline of atlas-based volumetry on Matlab (R2020b, Mathworks, Natick, Massachusetts) using SPM12 (Wellcome Trust Centre for Neuroimaging, London, UK; [www.fil.ion.ucl.ac.uk/spm](http://www.fil.ion.ucl.ac.uk/spm)) as described in previous studies.^6-7^ Brain, grey matter (GM), white matter (WM), cerebrospinal fluid (CSF) and intracranial volumes (ICV) were determined by the “tissue volumes” utility of SPM12. Different probabilistic brain atlases were employed to calculate regional volumes, since not all regions are comprised in a single atlas. The Harvard-Oxford atlas of subcortical structures^8-11^ distributed with the Oxford Centre for Functional MRI of the Brain Software Library (FSL) package was employed for hippocampus, amygdala, caudate, putamen, nucleus accumbens, pallidum, and thalamus; the Neuromorphometrics atlas (<https://www.neuromorphometrics.com>) for ventral diencephalon and ventricles (lateral ventricles, inferior lateral ventricles, third ventricle, fourth ventricle); the JHU DTI-based white-matter atlas^12-13^ for inferior, middle and superior cerebellar peduncles; the basal ganglia atlas (https://www.nitrc.org/frs/?group_id=653)^14^ for substantia nigra, nucleus subthalamicus and red nucleus; the LONI Probabilistic Brain Atlas 40 (LPB)^15^ for brainstem structures, cortical lobes and cerebellum. For midbrain, pons, and medulla oblongata, the LPB brainstem mask was divided by two cutting planes, the first one passing through the superior pontine notch and the inferior edge of the quadrigeminal plate, the second one parallel to the first plane and through the inferior pontine notch, as previously described.

For regional volumes, the GM and WM volumes were summed. Also, volumes were summed (left + right volumes) for bilateral regions. All volumetric data were normalized to the intracranial volume (ICV) as performed in previous studies,^6-7^ as follows:

*corrected volume = (raw volume / subject ICV) * mean ICV of age-matched HC group*

**Statistical analyses**

R studio (R version 4.1.2) was used for statistical analyses. Comparisons between groups were performed with Fisher’s-exact-test, Wilcoxon-rank-sum-test ANOVA or Kruskal-Wallis rank sum test, as appropriate. P values of between-group comparisons were Bonferroni corrected.

Clinical progression was measured as annualised PSPRS absolute or percentage change, calculated as follows:

*Annualised PSPRS absolute* *change = (PSPRS_f_ – PSPRS_b_) / t*

*Annualised PSPRS percentage change = {[(PSPRS_f_ – PSPRS_b_) / PSPRS_b_] * 100} / t*

where PSPRS_f_ and PSPRS_b_ are the PSP rating scale values at follow-up and at baseline, respectively, and *t* is the inter-visit time interval in years.

Associations among baseline clinical or imaging variables and clinical progression were investigated using linear regression models, with age and sex as covariates. Regression coefficients, R-squared (R2) values and p values adjusted according to False Discovery Rate (FDR) correction were evaluated.

**Machine learning models**

After investigating the group associations using linear regression models, we employed machine learning technology aiming to predict longitudinal clinical progression at the individual level using baseline clinical and/or radiological data. Two well-known algorithms were used, namely Support Vector Machine (SVM) with RBF kernel,^19^ a method that allows the model to capture complex nonlinear relationships in the data, and Random Forest (RF), a method based on classification and regression trees (CART) constructing multiple trees and making predictions through a majority voting mechanism.^21^ The machine learning analysis was performed with Python 3.11.7 and the package scikit-learn v1.2.2. The analyses were performed using two alternative approaches: a regression task aiming to predict the individual annualised PSPRS total score change, and a classification task aiming to distinguish “fast progressors” from “slow progressors” (stratified by the median annualised PSPRS absolute or percentage change values). In the regression tasks, Support Vector Regression (SVR) and Random Forest Regression (RFR) algorithms were employed, and the R-squared (R2) and mean absolute error (MAE) were extracted as performance metrics. For the classification task, the SVM and Random Forest (RF) were employed and the area under the receiver operating characteristic curve (AUC-ROC) was extracted as performance metric. Clinical and MRI volumetric data used as input for machine learning models were scaled to a similar range using *StandardScaler* due to the large discrepancy in magnitude values across predictors. The predictors included data which were available for all patients; thus, there were no missing data in the dataset. Subsequently, we employed a robust 5-fold stratified nested cross-validation procedure to evaluate the models’ performances avoiding data leakage. In brief, the outer cross-validation loop splits the dataset into training and testing sets (80%-20%) to provide an unbiased estimate of model’s performance on unseen data, while the inner loop performed hyperparameter optimization using *RandomizedSearchCV* (n_iter = 10) to maximize accuracy. Both the inner and outer fold sample sizes were based on an 80-20 split (80% for training and 20% for testing). This means that each fold of the outer loop included 20% of the whole sample (309/5 = 62 subjects); the inner loop was performed on the 80%-training group (n=247) with the same procedure and 80-20 split, thus each fold in the inner loop included 247/5= 49 subjects. In the classification tasks, the cross-validation procedure was “stratified” (StratifiedKFold) both in the inner and outer folds ensuring that balance between classes (fast and slow progressors) was preserved in each fold. For SV algorithm, tuned hyperparameters were C and gamma. For RF algorithm, tuned hyperparameters were: the number of trees, the maximum depth of each tree, the minimum number of samples required to split an internal node, and the minimum number of samples required to be at a leaf node. In detail, the hyperparameter space for the SVM classifier included C values in [0.001, 0.01, 0.1, 1, 10, 100, 1000] and gamma in [0.0001, 0.001, 0.01, 0.1, 1, 10], resulting in 42 possible parameter combinations. RandomizedSearchCV with n_iter=10 was employed to randomly sample 10 combinations for tuning in each trial. The hyperparameter space for the Random Forest classifier included: n_estimators: [50, 100, 150, 200], max_depth: [5, 10, 15, 20], min_samples_split: [5, 10, 15], and min_samples_leaf: [2, 5, 10], resulting in 144 possible parameter combinations. The same hyperparameter space was used for classifiers and regressors. The whole cross-validation procedure was repeated 10 times to increase performance reliability; the mean values of performance metrics and their standard deviations across outer folds and repetitions (5 folds × 10 repetitions) were calculated for each model.

**Supplementary References**

1. Dam T, Boxer AL, Golbe LI, et al; PASSPORT Study Group. Safety and efficacy of anti-tau monoclonal antibody gosuranemab in progressive supranuclear palsy: a phase 2, randomized, placebo-controlled trial. *Nat Med*. 2021; 27(8):1451-1457. doi: 10.1038/s41591-021-01455-x.
2. Boxer AL, Lang AE, Grossman M, et al; AL-108-231 Investigators. Davunetide in patients with progressive supranuclear palsy: a randomised, double-blind, placebo-controlled phase 2/3 trial. *Lancet Neurol*. 2014; 13(7):676-85. doi: 10.1016/S1474-4422(14)70088-2.
3. Höglinger GU, Litvan I, Mendonca N, et al. Arise Investigators. Safety and efficacy of tilavonemab in progressive supranuclear palsy: a phase 2, randomised, placebo-controlled trial. Lancet Neurol. 2021; 20(3):182-192. doi: 10.1016/S1474-4422(20)30489-0.
4. Höglinger GU, Huppertz HJ, Wagenpfeil S, et al; TAUROS MRI Investigators. Tideglusib reduces progression of brain atrophy in progressive supranuclear palsy in a randomized trial. *Mov Disord*. 2014; 29(4):479-87. doi: 10.1002/mds.25815.
5. Respondek G, Höglinger GU. DescribePSP and ProPSP: German Multicenter Networks for Standardized Prospective Collection of Clinical Data, Imaging Data, and Biomaterials of Patients With Progressive Supranuclear Palsy. *Front Neurol*. 2021; 12:644064. doi: 10.3389/fneur.2021.644064.
6. Quattrone A, Franzmeier N, Huppertz HJ, et al. Magnetic Resonance Imaging Measures to Track Atrophy Progression in Progressive Supranuclear Palsy in Clinical Trials. Mov Disord. 2024; 39(8):1329-1342. doi: 10.1002/mds.29866.
7. Höglinger GU, Schöpe J, Stamelou M, et al; AL-108-231 Investigators; Tauros MRI Investigators; Movement Disorder Society-Endorsed PSP Study Group. Longitudinal magnetic resonance imaging in progressive supranuclear palsy: A new combined score for clinical trials. *Mov Disord.* 2017; 32(6):842-852. doi: 10.1002/mds.26973.
8. Frazier JA, Chiu S, Breeze JL, et al. Structural brain magnetic resonance imaging of limbic and thalamic volumes in pediatric bipolar disorder. *Am J Psychiatry*. 2005; 162(7):1256-65. doi: 10.1176/appi.ajp.162.7.1256.
9. Desikan RS, Ségonne F, Fischl B, et al. An automated labeling system for subdividing the human cerebral cortex on MRI scans into gyral based regions of interest. *Neuroimage.* 2006;31(3):968-80. doi: 10.1016/j.neuroimage.2006.01.021.
10. Makris N, Goldstein JM, Kennedy D, et al. Decreased volume of left and total anterior insular lobule in schizophrenia. *Schizophr Res.* 2006; 83(2-3):155-71. doi: 10.1016/j.schres.2005.11.020.
11. Goldstein JM, Seidman LJ, Makris N, et al. Hypothalamic abnormalities in schizophrenia: sex effects and genetic vulnerability. *Biol Psychiatry*. 2007; 61(8):935-45. doi: 10.1016/j.biopsych.2006.06.027.
12. Mori S, Oishi K, Jiang H, et al. Stereotaxic white matter atlas based on diffusion tensor imaging in an ICBM template. *Neuroimage*. 2008; 40(2):570-582. doi: 10.1016/j.neuroimage.2007.12.035.
13. Oishi K, Zilles K, Amunts K, et al. Human brain white matter atlas: identification and assignment of common anatomical structures in superficial white matter. *Neuroimage*. 2008; 43(3):447-57. doi: 10.1016/j.neuroimage.2008.07.009
14. Keuken MC, Bazin PL, Backhouse K, et al. Effects of aging on T1, T2*, and QSM MRI values in the subcortex. Brain Structure and Function 2017; 222(6):2487-2505. doi: 10.1007/s00429-016-1352-4
15. Shattuck DW, Mirza M, Adisetiyo V, et al. Construction of a 3D probabilistic atlas of human cortical structures. *Neuroimage.* 2008; 39(3):1064-80. doi: 10.1016/j.neuroimage.2007.09.031.
