# Supplementary material for: Brain Atrophy Does Not Predict Clinical Progression in Progressive Supranuclear Palsy
Source: Mov Disord. 2025 Aug 30;40(11):2517–30. doi: 10.1002/mds.70026 (PMC12661634; doi:10.1002/mds.70026)
Supplement: Supplementary file 7 — Supplementary Table S1. Demographic and imaging data of patients with progressive supranuclear palsy and control subjects. [file MDS-40-2517-s002.docx]

| **Data** | **PSP-RS patients**  **(N=309)** | **Healthy Controls**  **(N=258)** | **p value** | **Mean PSP**  **difference to HC** | **Mean PSP**  **z-score to HC** |
| --- | --- | --- | --- | --- | --- |
| Sex, (M/F) | 161 / 148 | 133 / 125 | 0.933^a^ | / | / |
| Age at MRI (years) | 68.8 ± 6.5 | 68.2 ± 8.2 | 0.559^b^ | / | / |
|  |  |  |  |  |  |
| ***MR volumetry*** |  |  |  |  |  |
| Brain volume | 990 ± 60 | 1038 ± 49 | **<0.001^c^** | -4.6% | -0.98 |
| GM volume | 587 ± 50 | 610 ± 41 | **<0.001^c^** | -3.7% | -0.56 |
| WM volume | 403 ± 38 | 428 ± 33 | **<0.001^c^** | -5.9% | -0.75 |
| CSF volume | 409 ± 60 | 361 ± 49 | **<0.001^c^** | +13.3% | +0.98 |
| Intracranial volume | 1367 ± 137 | 1403 ± 123 | **<0.001^c^** | -2.5% | -0.29 |
| Frontal lobe | 271 ± 22 | 292 ± 20 | **<0.001^c^** | -7.4% | -1.01 |
| Temporal lobe | 176 ± 13 | 180 ± 10 | **<0.001^c^** | -2.5% | -0.44 |
| Parietal lobe | 158 ± 13 | 162 ± 10 | **<0.001^c^** | -2.6% | -0.39 |
| Occipital lobe | 115 ± 9 | 115 ± 8 | 0.203^c^ | +0.5% | +0.08 |
| Insula | 15.6 ± 1.4 | 16.1 ± 1.4 | **<0.001^c^** | -3.1% | -0.36 |
| Brainstem | 26.5 ± 2.7 | 30.3 ± 2.1 | **<0.001^c^** | -12.5% | -1.83 |
| Midbrain | 8.54 ± 0.84 | 10.20 ± 0.61 | **<0.001^c^** | -16.3% | -2.70 |
| Pons | 13.8 ± 1.6 | 15.6 ± 1.3 | **<0.001^c^** | -11.3% | -1.37 |
| Medulla | 4.20 ± 0.42 | 4.57 ± 0.35 | **<0.001^c^** | -8.0% | -1.04 |
| Cerebellum | 106 ± 11 | 112 ± 8 | **<0.001^c^** | -5.4% | -0.73 |
| Lateral ventricles | 36.9 ± 15.7 | 25.8 ± 10.5 | **<0.001^c^** | +42.7% | +1.05 |
| Inferior lateral ventricles | 0.43 ± 0.29 | 0.29 ± 0.14 | **<0.001^c^** | +44.7% | +0.92 |
| Third ventricle | 1.45 ± 0.45 | 1.03 ± 0.33 | **<0.001^c^** | +41.8% | +1.28 |
| Fourth ventricle | 1.78 ± 0.53 | 1.44 ± 0.33 | **<0.001^c^** | +24.1% | +1.04 |
| MCP | 9.88 ± 1.15 | 10.91 ± 0.90 | **<0.001^c^** | -9.5% | -1.14 |
| SCP | 1.12 ± 0.14 | 1.32 ± 0.10 | **<0.001^c^** | -15.3% | -1.94 |
| ICP | 1.20 ± 0.12 | 1.33 ± 0.10 | **<0.001^c^** | -9.8% | -1.32 |
| Caudate nucleus | 3.84 ± 0.66 | 4.33 ± 0.56 | **<0.001^c^** | -11.2% | -0.86 |
| Putamen | 5.64 ± 0.78 | 6.11 ± 0.82 | **<0.001^c^** | -7.6% | -0.57 |
| Pallidum | 3.19 ± 0.32 | 3.76 ± 0.27 | **<0.001^c^** | -15.2% | -2.13 |
| Thalamus | 10.8 ± 1.2 | 11.7 ± 1.0 | **<0.001^c^** | -7.5% | -0.86 |
| Nucleus accumbens | 0.88 ± 0.11 | 0.96 ± 0.09 | **<0.001^c^** | -8.5% | -0.87 |
| Ventral Diencephalon | 6.85 ± 0.69 | 8.15 ± 0.53 | **<0.001^c^** | -15.6% | -2.44 |
| Red nucleus | 0.35 ± 0.06 | 0.47 ± 0.04 | **<0.001^c^** | -24.7% | -2.81 |
| Substantia nigra | 0.44 ± 0.06 | 0.54 ± 0.04 | **<0.001^c^** | -19.4% | -2.58 |
| Subthalamic nucleus | 0.05 ± 0.01 | 0.06 ± 0.01 | **<0.001^c^** | -24.0% | -3.00 |
| Hippocampus | 6.06 ± 0.73 | 6.29 ± 0.56 | **<0.001^c^** | -3.7% | -0.42 |
| Amygdala | 3.37 ± 0.42 | 3.48 ± 0.30 | **<0.001^c^** | -3.14% | -0.36 |

**Supplementary Table 1.** Demographic and imaging data of patients with progressive supranuclear palsy and control subjects.

Abbreviations: PSP-RS = progressive supranuclear palsy – Richardson’s Syndrome; GM = grey matter; WM = white matter; CSF = cerebrospinal fluid; SCP = superior cerebellar peduncle; MCP = middle cerebellar peduncle; ICP = inferior cerebellar peduncle.

Data are shown as mean ± standard deviation; volume results are shown in ml; all volumes were normalized to the mean intracranial volume of controls. Significant p values after Bonferroni correction (p<0.05/34; p<0.0014). are highlighted in bold.

^a^Fisher’s exact test. ^b^Wilcoxon rank sum test. ^c^ANCOVA with age and sex as covariates. A color scale was used to highlight and rank the most involved regions in PSP compared to HC, with shades of red (lower numbers or volume loss) and shades of blue (higher numbers or volume increase).
