# Supplementary material for: Brain Atrophy Does Not Predict Clinical Progression in Progressive Supranuclear Palsy
Source: Mov Disord. 2025 Aug 30;40(11):2517–30. doi: 10.1002/mds.70026 (PMC12661634; doi:10.1002/mds.70026)
Supplement: Supplementary file 8 — Supplementary Table S2. Associations between regional brain volumes and clinical severity at baseline in patients with progressive supranuclear palsy. [file MDS-40-2517-s011.docx]

**Supplementary Table 2.** Associations between regional brain volumes and clinical severity at baseline in patients with progressive supranuclear palsy.

| **Imaging Data** | **Coefficient** | **Beta value** | **Adjusted**  **R-squared** | **p value** | **Adjusted P Value** |
| --- | --- | --- | --- | --- | --- |
| Brain | -0.038 | -0.225 | 0.04 | < 0.001 | **0.002** |
| Gray Matter | -0.023 | -0.109 | 0.01 | 0.097 | 0.121 |
| White Matter | -0.041 | -0.151 | 0.02 | 0.010 | **0.015** |
| CSF | 0.038 | 0.225 | 0.04 | < 0.001 | **0.002** |
| Intracranial Volume | -0.015 | -0.197 | 0.03 | 0.007 | **0.012** |
| Frontal lobe | -0.077 | -0.166 | 0.03 | 0.009 | **0.014** |
| Temporal lobe | -0.067 | -0.083 | 0.01 | 0.213 | 0.248 |
| Parietal lobe | -0.071 | -0.087 | 0.01 | 0.174 | 0.210 |
| Occipital lobe | -0.050 | -0.043 | 0.00 | 0.513 | 0.544 |
| Insula | -0.359 | -0.049 | 0.00 | 0.428 | 0.469 |
| Brainstem | -1.300 | -0.340 | 0.12 | < 0.001 | **< 0.001** |
| Midbrain | -3.887 | -0.317 | 0.10 | < 0.001 | **< 0.001** |
| Pons | -2.029 | -0.321 | 0.11 | < 0.001 | **< 0.001** |
| Medulla | -7.619 | -0.316 | 0.09 | < 0.001 | **< 0.001** |
| Cerebellum | -0.210 | -0.214 | 0.04 | < 0.001 | **0.001** |
| Ventral Diencephalon | -4.419 | -0.296 | 0.09 | < 0.001 | **< 0.001** |
| Lateral ventricle | 0.155 | 0.236 | 0.05 | < 0.001 | **< 0.001** |
| Inferior Lateral ventricle | 3.675 | 0.102 | 0.01 | 0.092 | 0.119 |
| Third ventricle | 7.598 | 0.336 | 0.10 | < 0.001 | **< 0.001** |
| Fourth ventricle | 5.538 | 0.288 | 0.08 | < 0.001 | **< 0.001** |
| Middle cerebellar peduncle | -2.263 | -0.252 | 0.06 | < 0.001 | **< 0.001** |
| Inferior cerebellar peduncle | -26.021 | -0.314 | 0.10 | < 0.001 | **< 0.001** |
| Superior cerebellar peduncle | -27.781 | -0.369 | 0.14 | < 0.001 | **< 0.001** |
| Hippocampus | -0.309 | -0.022 | 0.00 | 0.732 | 0.753 |
| Amygdala | -1.419 | -0.057 | 0.01 | 0.353 | 0.398 |
| Caudate | -3.377 | -0.218 | 0.05 | < 0.001 | **< 0.001** |
| Putamen | -1.899 | -0.144 | 0.02 | 0.014 | **0.019** |
| Nucleus Accumbens | -14.466 | -0.156 | 0.02 | 0.011 | **0.015** |
| Pallidum | -6.881 | -0.216 | 0.05 | < 0.001 | **< 0.001** |
| Thalamus | -2.242 | -0.253 | 0.05 | < 0.001 | **< 0.001** |
| Red nucleus | -50.521 | -0.287 | 0.09 | < 0.001 | **< 0.001** |
| Substantia nigra | -50.744 | -0.286 | 0.08 | < 0.001 | **< 0.001** |
| Nucleus subthalamicus | -358.323 | -0.263 | 0.07 | < 0.001 | **< 0.001** |

Data obtained on the whole cohort of 309 Progressive supranuclear palsy-Richardson’s syndrome patients. Significant p values surviving FDR correction are highlighted in bold. All models included age and sex at baseline as covariates. A color scale was used to highlight and rank the strength of significant associations, with shades of red (negative associations) and shades of blue (positive associations). All models had adjusted R-squared values < 0.15, meaning the models explained less than 15% of variance of the baseline clinical severity.
