# Supplementary material for: Brain Atrophy Does Not Predict Clinical Progression in Progressive Supranuclear Palsy
Source: Mov Disord. 2025 Aug 30;40(11):2517–30. doi: 10.1002/mds.70026 (PMC12661634; doi:10.1002/mds.70026)
Supplement: Supplementary file 9 — Supplementary Table S3. Associations of baseline clinical and imaging data with longitudinal clinical progression in patients with progressive supranuclear palsy. [file MDS-40-2517-s006.docx]

**Supplementary Table 3.** Associations of baseline clinical and imaging data with longitudinal clinical progression in patients with progressive supranuclear palsy.

| **Data** | **Coefficient** | **Beta value** | **Adjusted**  **R-squared** | **p value** | **FDR Adjusted**  **p value** |
| --- | --- | --- | --- | --- | --- |
| *Annualised PSPRS total score absolute change* |  |  |  |  |  |
| Brain | -0.041 | -0.280 | 0.07 | < 0.001 | **< 0.001** |
| Grey Matter | -0.036 | -0.203 | 0.04 | 0.002 | **0.013** |
| CSF | 0.041 | 0.280 | 0.07 | < 0.001 | **< 0.001** |
| Frontal Lobe | -0.138 | -0.343 | 0.11 | < 0.001 | **< 0.001** |
| Parietal Lobe | -0.162 | -0.229 | 0.05 | < 0.001 | **0.003** |
| Superior cerebellar peduncle | -9.832 | -0.151 | 0.03 | 0.003 | **0.036** |
| Inferior Lateral Ventricle | 5.968 | 0.192 | 0.04 | 0.001 | **0.012** |
| Third ventricle | 3.438 | 0.175 | 0.04 | 0.004 | **0.025** |
| Fourth ventricle | 2.914 | 0.175 | 0.04 | 0.003 | **0.017** |
|  |  |  |  |  |  |
| *Annualised PSPRS total score percentage change* |  |  |  |  |  |
| PSPRS total score | -0.890 | -0.326 | 0.12 | < 0.001 | **< 0.001** |
| PSPRS history score | -2.133 | -0.241 | 0.07 | < 0.001 | **< 0.001** |
| PSPRS mentation score | -1.581 | -0.142 | 0.03 | 0.012 | **0.046** |
| PSPRS bulbar score | -3.704 | -0.195 | 0.05 | 0.001 | **0.003** |
| PSPRS ocular score | -1.775 | -0.193 | 0.05 | 0.001 | **0.003** |
| PSPRS limb score | -2.860 | -0.204 | 0.06 | < 0.001 | **0.002** |
| PSPRS gait and midline score | -1.925 | -0.255 | 0.08 | < 0.001 | **< 0.001** |
| Brain | -0.084 | -0.181 | 0.04 | 0.008 | **0.032** |
| CSF | 0.084 | 0.181 | 0.04 | 0.008 | **0.032** |
| Frontal lobe | -0.333 | -0.262 | 0.07 | < 0.001 | **< 0.001** |
| Parietal lobe | -0.409 | -0.184 | 0.04 | 0.004 | **0.019** |

Abbreviations: PSP = progressive supranuclear palsy; PSPRS = PSP rating scale.

Data obtained on the whole cohort of 309 Progressive supranuclear palsy-Richardson’s syndrome patients. Significant p values surviving FDR correction are highlighted in bold. Only significant associations after FDR correction are shown in the table; the full list of tested baseline variables (n=40) included PSPRS total score and categories’ scores, and the imaging variables shown in supplementary table 1. All models included age and sex at baseline as covariates. A color scale was used to highlight and rank the strength of significant associations, with shades of red (negative associations) and shades of blue (positive associations). All models with the following structure (response ~ predictor + age + sex) had adjusted R-squared values < 0.15, meaning the models explained less than 15% of variance of clinical progression rate. All associations between baseline imaging variables and subsequent one-year clinical progression were confirmed when baseline PSPRS score was included as covariate.
