# Supplementary material for: Brain Atrophy Does Not Predict Clinical Progression in Progressive Supranuclear Palsy
Source: Mov Disord. 2025 Aug 30;40(11):2517–30. doi: 10.1002/mds.70026 (PMC12661634; doi:10.1002/mds.70026)
Supplement: Supplementary file 10 — Supplementary Table S4. Explained interpatient variance of clinical progression by multivariate linear regression modeling using clinical and/or imaging data. [file MDS-40-2517-s008.docx]

**Supplementary Table 4.** Explained inter-patient variance of clinical progression by multivariate linear regression modeling using clinical and/or imaging data

| **Data** | **Number of predictors** | **Adjusted R2 value** | **p value** |
| --- | --- | --- | --- |
| *Annualised PSPRS total score absolute change* |  |  |  |
| Clinical predictors | 9 | 0.006 | 0.289 |
| Imaging predictors | 35 | 0.143 | **<0.001** |
| Clinical and imaging predictors | 42 | 0.153 | **<0.001** |
|  |  |  |  |
| *Annualised PSPRS total score percentage change* |  |  |  |
| Clinical predictors | 9 | 0.114 | **< 0.001** |
| Imaging predictors | 35 | 0.092 | **0.002** |
| Clinical and imaging predictors | 42 | 0.219 | **< 0.001** |

Abbreviations: PSP = progressive supranuclear palsy; PSPRS = PSP rating scale.

Data obtained on the whole cohort of 309 Progressive supranuclear palsy-Richardson’s syndrome patients. Most linear models showed statistically significant ability to explain inter-patient variability in longitudinal change of PSPRS total score but had low R2 values. For interpretation, an adjusted R2 value of 0.219 means that the model was able to explain 21.9% of inter-individual variance in the PSPRS total score change.
