# Supplementary material for: Brain Atrophy Does Not Predict Clinical Progression in Progressive Supranuclear Palsy
Source: Mov Disord. 2025 Aug 30;40(11):2517–30. doi: 10.1002/mds.70026 (PMC12661634; doi:10.1002/mds.70026)
Supplement: Supplementary file 11 — Supplementary Table S5. Classification performance of machine learning models in differentiating fast from slow progressors in the validation folds. [file MDS-40-2517-s010.docx]

**Supplementary Table 5.** Classification performance of machine learning models in differentiating fast from slow progressors in the validation folds.

| **Data** | **Number of predictors** | **SVM** | **RF** |
| --- | --- | --- | --- |
| *Annualised PSPRS total score absolute change* |  |  |  |
| Clinical predictors | 9 | 0.506 ± 0.023 | 0.528 ± 0.021 |
| Imaging predictors | 35 | 0.616 ± 0.019 | 0.633 ± 0.012 |
| Clinical and imaging predictors | 42 | 0.609 ± 0.024 | 0.637 ± 0.021 |
|  |  |  |  |
| *Annualised PSPRS total score percentage change* |  |  |  |
| Clinical predictors | 9 | 0.687 ± 0.009 | 0.668 ± 0.012 |
| Imaging predictors | 35 | 0.544 ± 0.025 | 0.554 ± 0.026 |
| Clinical and imaging predictors | 42 | 0.674 ± 0.025 | 0.678 ± 0.024 |

Abbreviations: PSP = progressive supranuclear palsy; PSPRS = PSP rating scale; SVM = Support Vector Machine; RF = Random Forest. Data obtained on the whole cohort of 309 Progressive supranuclear palsy-Richardson’s syndrome patients. The table shows mean ± standard deviation of Area under the receiver operating characteristic curve (AUC-ROC) values in distinguishing fast progressors from slow progressors in the validation folds in stratified nested cross-validation procedure, repeated 10 times. Fast progressors were patients with annualised PSPRS change values above the median; slow progressors were patients with annualised PSPRS change values below the median value. For annualised PSPRS total score absolute change, the median value in the whole cohort of 309 PSP patients was: 10.03 points; for the annualised PSPRS total score percentage change, the median value was: 26.74%.
